# Benefits and applications of microwave-assisted synthesis of nitrogen containing heterocycles in medicinal chemistry

**DOI:** 10.1039/d0ra01378a

**Published:** 2020-04-07

**Authors:** Maged Henary, Carl Kananda, Laura Rotolo, Brian Savino, Eric A. Owens, Giancarlo Cravotto

**Affiliations:** Department of Chemistry, Georgia State University 100 Piedmont Ave SE Atlanta Georgia 30303 USA mhenary1@gsu.edu +1 404 413-5566; Center for Diagnostics and Therapeutics, Georgia State University 100 Piedmont Ave SE Atlanta Georgia 30303 USA; Department of Drug Science and Technology and NIS – Centre for Nanostructured Interfaces and Surfaces, University of Turin via P. Giuria 9 10125 Turin Italy

## Abstract

Nitrogen containing heterocycles are of immense research interest because they are often found as naturally occurring bioactive compounds. The prominence of N-heterocycles makes it vital to develop methods to increase their synthetic efficiencies and probe the effects of their modifications on biological efficacy. Medicinal chemists have exploited microwave-assisted organic synthesis (MAOS) to facilitate the development of complex heterocyclic structures. MAOS is a growing synthetic methodology among medicinal chemists and has proven to be more efficient in terms of reaction yield, reaction time, product purity and environmental friendliness for many reactions when compared to conventional thermal methods for cycloaddition and selective functionalization. The importance of nitrogen containing ring systems in medicine cannot be understated, as such ring systems have shown to be applicable in compounds such as vitamins, herbicides, anti-fungal agents, anti-bacterial agents and anti-cancer agents, among other things. The significance of these applications has created an unprecedented need for more efficient synthetic methods. This review presents an overview of MAOS and its role in recent and pressing advancements for the synthesis of small- and medium-sized nitrogen containing heterocycles, including pyrroles, indoles, pyridines, pyrrolidines, imidazoles, pyrazoles, pyrazolines, lactams, and 1,2,3-triazoles, which are significant scaffolds for compounds with medicinal uses.

## Abstract 2

Nitrogen containing heterocycles are of immense research interest because they are often found as naturally occurring bioactive compounds.

## Graphical abstract

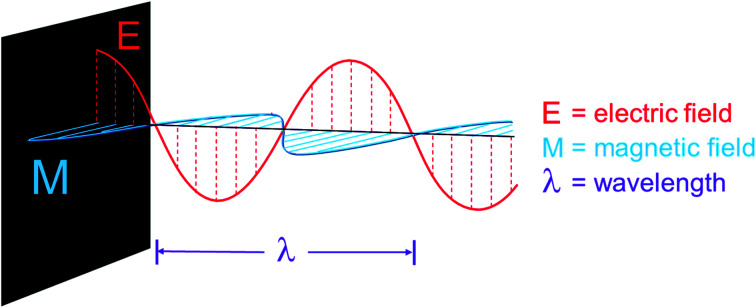

## 1. Introduction

Heterocyclic ring systems have attracted a great deal of attention due to their reoccurrence in many biologically active molecules. A brief survey of the most active pharmacophores shows that nitrogen-based heterocycles are the most prevalent form of biologically relevant small molecules.^1^ To date, N-heterocycles remain scaffolds for compounds that exhibit interesting biological activities and are used in many different pharmacological areas.^2^ Such ring systems have various applications, ranging from vitamins and herbicides to anti-fungal, anti-bacterial and anti-cancer agents. The development of more efficient synthetic procedures has been an ongoing quest.^3^ In the past few years, synthetic chemists have developed various methods for the preparation of heterocyclic compounds using expensive palladium, gold or other equally expensive and environmentally polluting catalysts.^4^ In this context, microwave (MW) irradiation is particularly beneficial in biologically important N-heterocycle preparation, leading to high yield with low environmental impact.^5*a*–*c*^

**Fig. 1:**
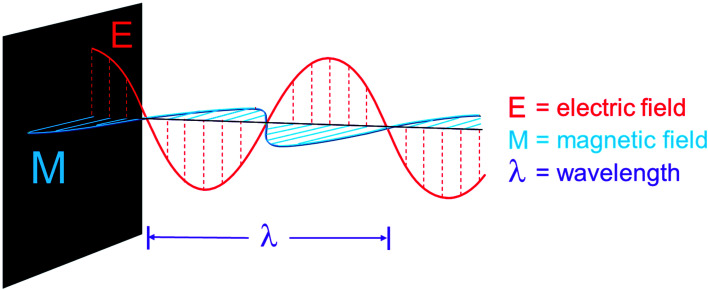
General schematic for microwave energy.

Microwave-assisted organic synthesis (MAOS) exploits dielectric volumetric heating as an alternative heat source, which results in faster and more selective reactions due to the uniform heat distribution. This “superman heat vision” effect is based on temperature increase by dielectric heating, which occurs through two means: dipolar polarization and ionic conduction. Microwave dielectric heating drives chemical reactions by taking advantage of the ability of the medium to channel electromagnetic radiation into heat – this happens when the dipoles or ions present in the reaction mixture align in an applied electric field as a result to MW irradiation. As the electric field oscillates, the dipoles or ion field attempt to realign with the oscillating electric field and, in the process, lose energy in the form of heat due to molecular friction and dielectric losses.^6^ MW irradiation can freely pass through the walls of the reaction vessel, coupling directly with molecules and ions of the reaction mixture. Thus, the tendency for the reaction mixture to boil is reduced. In fact, superheating above the boiling point of the solvent is known to occur under MW conditions. Since dielectric heating is only produced within an absorbing material that converts MW energy into heat, reactions vials have to be transparent to the electromagnetic waves. There are many studies about the correlation between the penetration depth as a function of temperature and MW frequency, and it has been shown that generally, materials with a low loss tangent have high penetration depths.^7^ A properly designed cavity allows the temperature increase to be uniform throughout the sample, leading to a reduction in reaction time – typically from days or hours to minutes or seconds – and an increase in product yields, purity and atom efficiency if the correct conditions are optimized. Additionally, these benefits lend themselves directly to the production of medicinal compounds in an environmentally friendly way (Fig. 1).

MW instrumentation typically operates at a frequency of 2.45 GHz and is generally classified in two categories: monomode (or single-mode) and multi-mode MW reactors.^8^ The differentiating feature of a single-mode apparatus is its ability to create a standing wave pattern, which is generated by the interference of fields that have the same amplitude but different oscillating directions. This interface generates an array of nodes where MW energy intensity is zero and a collection of antinodes where the magnitude of MW energy is at a maximum.^9^ In the much smaller monomode cavities, the electromagnetic irradiation is directed through an accurately designed rectangular or circular wave guide onto the reaction vessel, which is mounted at a fixed distance from the magnetron. This distance should ensure that the sample is placed at the antinodes of the standing electromagnetic wave pattern.^10^

Monomode reactors are specifically designed for small-scale synthesis with applications in R&D laboratories, and can process under sealed-vessel conditions (volumes ranging from 0.2 to about 50 mL with temperatures up to 250 °C and pressures of 20 bar) or under open-vessel reflux conditions (volumes around 150 mL). Monomode MW heating equipment is currently applied in synthetic organic chemistry in solvent-free reactions, small-scale drug discovery development and optimization of methods.^11^

The most important advantage of single-mode apparatus is the high rate of heating, but, compared to multi-mode cavities, only one vessel can be irradiated at a time. However, after the completion of the reaction period, the reaction mixture can be rapidly cooled with compressed air. As a result, the apparatus becomes more user-friendly.^12^

Multi-mode apparatuses are conceptually the direct evolution of domestic ovens introduced to the US market in 1967. The MWs enter the cavity (around 40 L for multi-mode apparatuses) with relatively low field density and are then reflected by the walls and load in a rather chaotic manner. The goal is to generate as much disorder as possible inside the apparatus to avoid the generation of a standing wave pattern inside the oven cavity. The greater the chaos, the higher the dispersion of radiation, which increases the area of effective heating inside the apparatus.^13^ As a result, a multi-mode MW heating apparatus can accommodate a number of samples simultaneously for heating, and is thus the most useful method for industrial scale-up of processes considering that several grams to kilograms can be loaded in both open- and closed-vessel conditions.^14^ Some ovens even contain a double magnetron unit and are equipped with a pyramidal-shaped rotating diffuser, like the multi-mode MicroSynth and Milestone/MLS. A major limitation of the multi-mode oven is a lack of temperature uniformity due to the disorder generated. To optimize the homogeneity of the electromagnetic field distribution in the solvent, commercial ovens are equipped with a mode stirrer (SynthWave, Milestone) or use turntable reaction places (RotoSynth, Milestone) to ensure uniform conditions inside the cavity and minimize the difference between hot and cold spots created in the system. Dedicated MW reactors, such as pressurized ones, have extended the range of operating temperature, enabling the use of low boiling organic solvents and reagents.^15^ The last generation of professional MW reactors enables work in a wide range of temperatures (up to 300 °C) and pressures (up to 200 bar) and contains multiple gas inlets. Multiple racks can achieve a fast screening of reaction conditions by performing parallel tests and selecting the best solvent, catalyst, and ratio (SynthWave, Milestone).

Some successful examples of synthetic protocols from milligram screening to multigram preparation showed that the same dedicated MW reactors (batch or flow) are well suited for larger-scale production without further optimization studies.^16^

**Fig. 2:**
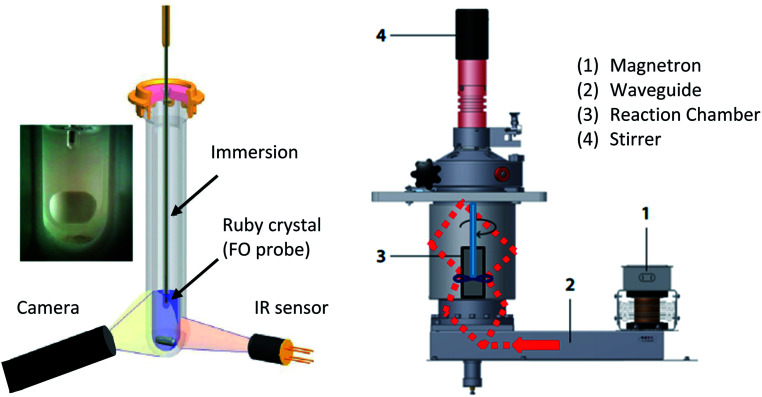
(Left): scheme of a monomode reactor.^17^ (Right): scheme of a multi-mode reactor.^16^

Recent research has resulted in the development of continuous-flow reactors for single- and multi-mode cavities that enable preparation of materials in kilogram scales (Fig. 2).

This review article presents the most recent and pressing advances made in MAOS for the formation of small and medium skeletally diverse N-heterocycles, and highlights examples of the judicious manipulation of selective parameters that influence the successful implementation of MW technology in chemical conversions. MAOS methods have been employed for maximizing the atom efficiency, yield, purity and scope for the synthesis of various heterocycles such as pyrroles, indoles, pyridines, pyrrolidines, imidazoles, pyrazoles, pyrazolines, lactams, and 1,2,3-triazoles, all structures with multiple relevant medicinal applications. In this article, representative samples of all discussed compounds are used to highlight the differences between conventional and microwave-assisted organic synthesis. We are confident that medicinal and organic chemists will take advantage of this survey toward N-heterocycle synthesis in drug discovery.

## 2. Pyrrole

There are many classic protocols for the synthesis of pyrroles, including the Knorr, Pall–Knorr, Hantzsch condensation and Clauson–Kaas reactions, which, under conventional heating, require harsh reaction and work-up conditions that partly offset product yield. New protocols under MW irradiation could dramatically improve pyrrole synthesis. Aydogan adopted MW heating in the Clauson–Kaas reaction of 2,5-dialkoxytetrahydrofurans 1 with various amines 2 in the presence of ionic liquid catalyst 1-hexyl-3-methylimidazolium hydrogen sulfate ([hmim][HSO_4_]) to yield N-substituted amines 3 (eqn (1)).^18^ In this homogeneous reaction, [hmim][HSO_4_] acted as both a solvent and promoter, interacting with MWs through the ionic conduction mechanism, enabling superheating and thus allowing for effective insertion of the amines into the ring in excellent yields (69–91%) without any significant product decomposition.1
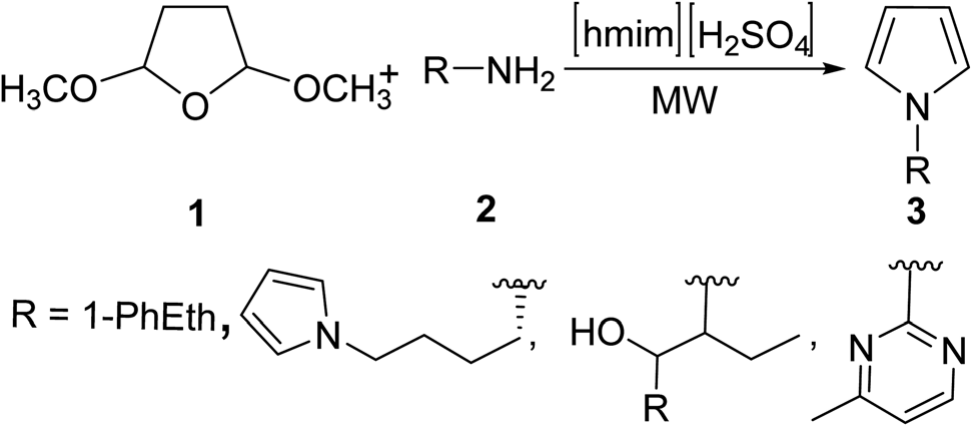


In order to broaden the scope of MW-assisted pyrrole synthesis, Mir *et al.*^19^ were able to equip various heterocycles on the pyrrole-3-methanol scaffold 6 using the MW irradiated reaction of succinaldehyde 4 and *N*-PMP-α-iminonitriles 5 (eqn (2)) in the presence of l-proline as a catalyst. DMSO, a polar (dielectric) organic solvent that was used in this approach, can absorb MW energy and enable effective heating of the reaction through the dielectric conversion of absorbed MWs into heat. Pyrrole analogs were obtained in good yields (50–75%) in less than one hour. 2-Substituted 3-hydroxymethyl pyrroles are not only important pharmacophores, but they also represent invaluable intermediates in the path to unlock the scaffold of a new class of bridged pyrrole heterocycles with immense therapeutic potential.2
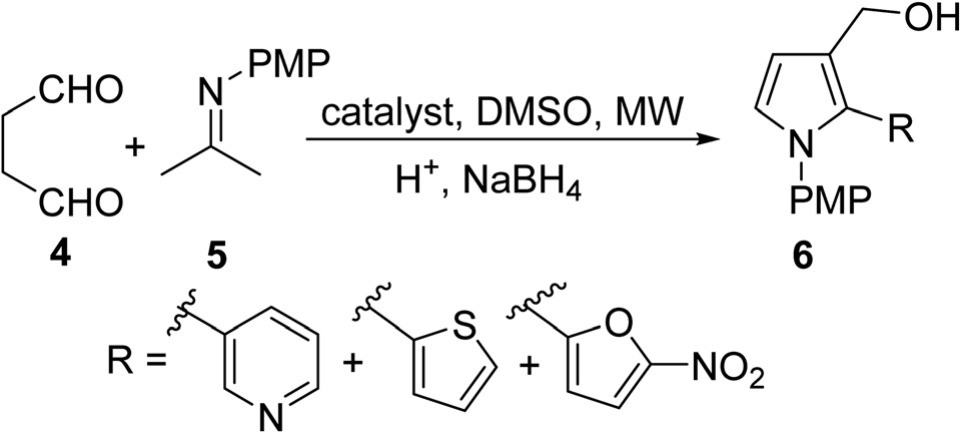


Lee *et. al.* succeeded in the synthesis of pyrroles bearing increased sites of substitution compared to previous examples through a Cu(ii)-promoted, one-pot, MW irradiation reaction of 1-alkynes 7 and primary amines 8 to yield *N*-2,5-trisubstituted pyrroles 9 (eqn (3)).^20^ The promotion of the following reaction under MW conditions can be directly attributed to the heterogeneous composition of the reaction system. The reaction is comprised of a highly polar and thus strongly MW-absorbing solvent, methanol, and a ferromagnetic metal catalyst, Cu, that can also strongly interact with the electromagnetic field. A “nonequilibrium local heating” phenomenon has been observed for comparable heterogeneous systems where the temperature inside the reaction vessel is governed by the dielectric loss of the polar solvent and by heat transfer from the ferromagnetic metal particles to the solvent.^21^ The following reaction allowed for the formation of alkyl and aryl functionalized pyrroles in good yield (42–82%) with short reaction times (<10 min).3
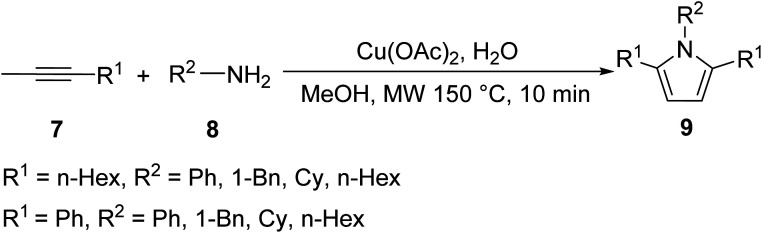


Wyrębek *et al.* investigated a zinc-catalyzed cyclization of homopropargyl azides 10 in the formation of N-unprotected fused cycloalkyl trisubstituted pyrroles 11 under MW irradiation (eqn (4)).^22^ Organic azides are sensitive reagents that tend to decompose under heating. Therefore, 1,2-dichloroethane (DCE), which absorbs less MWs compared to the methanol used in the previous example, may offer a more passive temperature increase under MW irradiation. In this way, the exothermal decomposition of the azide is avoided, leading to the complete conversion to the pyrrole. The following protocol allowed for the efficient formation of fused cycloalkyl groups on the pyrrole ring.4
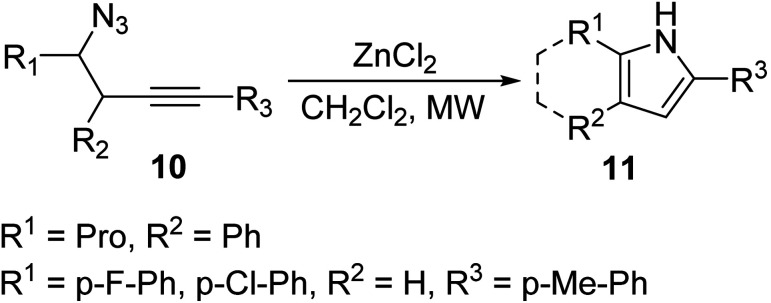


A variety of exotic tetra-substituted pyrrole derivatives 14 were synthesized by Bremner through the reaction of various secondary propargylamines 12 with aldehydes 13 under MW conditions (eqn (5)).^23^ In many reactions involving a large volume of a high boiling polar solvent such as DMF, removal of the solvent requires a complicated workup which leads to a yield reduction. Using MW heating in this example, only a small quantity of DMF was used and removed through a quick water wash. The following methodology makes it highly tolerable for bulky aliphatic groups and pharmaceutically relevant functionality such as nitrogen and sulfur heterocycles to be substituted on the pyrrole ring.5
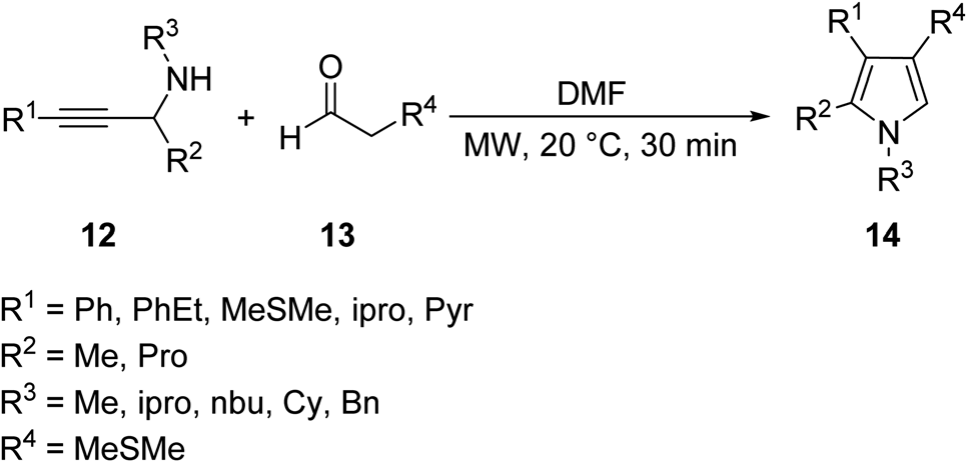


Zhang *et. al.*^24^ developed a copper-catalyzed MW assisted synthesis of fully substituted α-arylpyrroles 17 from β-enamino compounds 15 and propargyl acetates 16 (eqn (6)).6
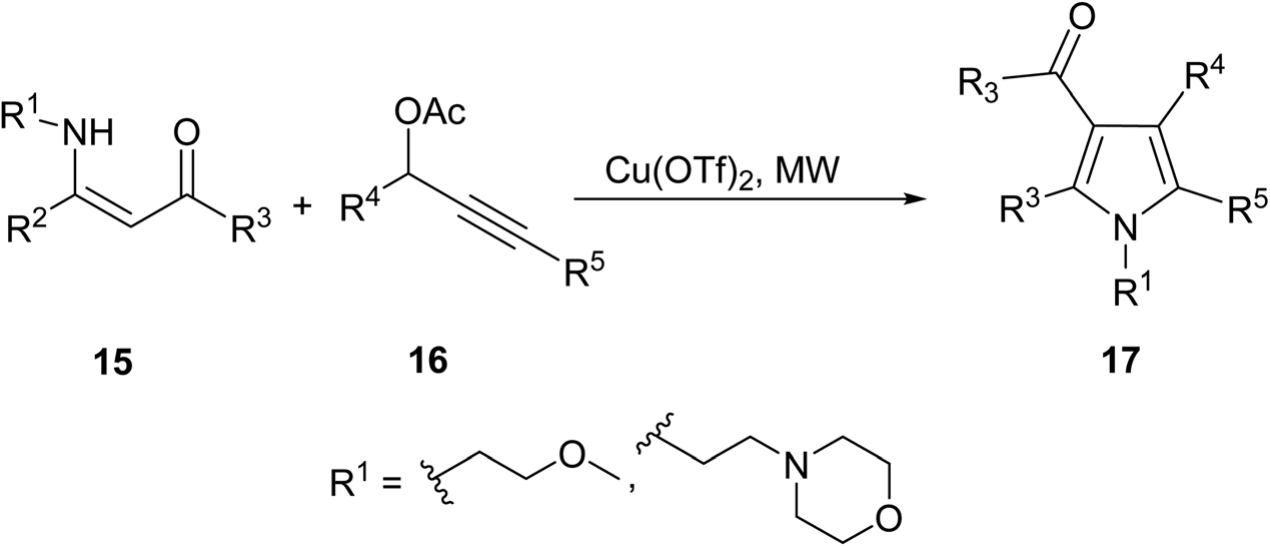


The synthesis of fully substituted α-arylpyrrole 17 was synthesized using a number of various conditions. The best conditions were those using a Cu(OTf)_2_ catalyst, and the results using conventional *versus* microwave techniques are highlighted in Table 1. One variation of compound 17 was used as a representative example. As can be seen, microwave heating resulted in a 25% higher yield with just one fifth the reaction time of conventional heating.^24^

**Table 1:** Conventional *versus* microwave methods in the synthesis of compound 17. In this case, R_1_ = Ph, R_2_ = Ph, R_3_ = COOEt, R_4_ = PhMeO, and R_5_ = H^24^

Method	Catalyst	Reaction time (min)	Yield (%)
Conventional (150 °C)	Cu(OTf)_2_	100	74
Microwave (150 °C)	Cu(OTf)_2_	20	99

### Medicinal applications

**Fig. 3:**
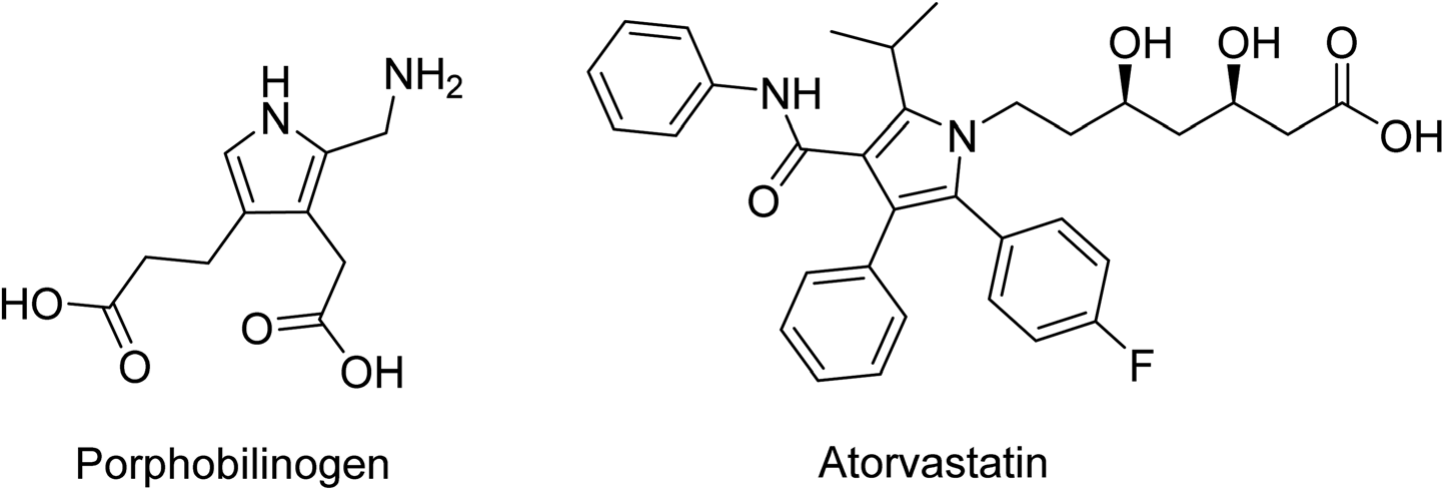
The chemical structures of Porphobilinogen and Atorvastatin.

The pyrrole ring, incorporated either as a substituent or with various substitutions on the ring itself, is among the most frequently observed heterocyclic systems in the structure of natural products and synthetic materials, probably because the electron characteristics on the ring are responsible for better binding with enzymes and receptors and lead to further modifications in the scaffold to attain an ideal activity profile.^25^ Some drugs containing a pyrrole moiety are already available on the market, while others are under clinical trials.^26^ Among the medicinal applications, pyrroles have pronounced antimycobacterial activities and some analogs show good therapeutic indexes. As reported by Surineni,^27^ a series of novel carbazole-tethered pyrrole derivatives with ferric chloride have a high antitubercular activity against *M. tuberculosis* H_37_Rv (MTB) with lower cytotoxicity profiles compared to other evaluated compounds. Antimicrobial activity can also be attained with new bis-pyrrole derivatives from hydrazonoyl halides.^28^ The results revealed that most of the tested compounds exhibit better activity against Gram-positive bacteria over Gram-negative bacteria (*Pseudomonas aeruginosa* and *Escherichia coli*), and one of them was found to be the most potent relative to the standard drug, Itraconazole, against *Aspergillus fumigates*. Atorvastatin, known commercially as Lipitor, is a drug used to treat high cholesterol, and is also a well-known pyrrole-containing derivative (Fig. 3).^29^ Furthermore, the blood respiratory pigment heme and photosynthesis pigment chlorophyll are biosynthesized from the pyrrole porphobilinogen.^30^ Additionally, pyrimidine–pyrrole appended triazoles were developed by Thiriveedhi *et. al.* and showed promise in their anti-cancer properties through tests against breast cancer and melanoma cell lines. With microwave synthesis, a number of these compounds could be rapidly synthesized and tested for anti-cancer activity, speeding up the process of finding novel and effective anti-cancer agents.^31^ Besides all these activities, pyrroles can show antiviral activity against viruses and tumors and show promise in anticoccidial, anti-inflammatory, antipsychotic, anticonvulsant and antitumor activities. Pyrroles are also inhibitors for histone deacetylase, CDK, Monoamine oxidase and EGFR tyrosine kinases.^32^

## 3. Indole

Halogenated indoles are important targets in the design of bioactive indoles because they can be equipped with varying moieties at the polarized carbon to outfit desired pharmacological output. Wang *et. al.*^33^ reported the synthesis of 2-haloindole 19*via* a metal-free cyclization of 2-(gem-dibromo vinyl)-*N*-methylsulfonylanilines 18 in the presence of tetra-butylammonium fluoride (TBAF) (eqn (7)). Strong electron-withdrawing groups can activate the amine^34^ and allow for the efficient intra-molecular cyclization of the linker to the corresponding halo-indole.7
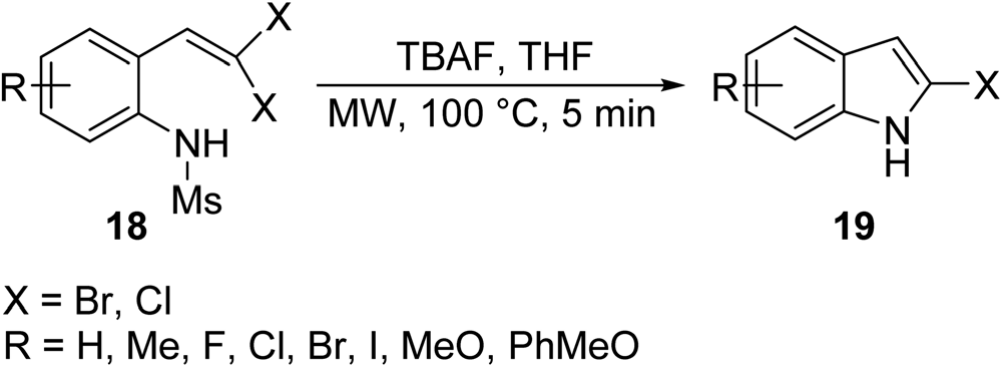


Nguyen *et. al.*^25^ synthesized functionalized 3-nitroindoles 21 bearing much more diverse electron withdrawing groups (EWG) in an effective and facile MW mediated intermolecular Heck cyclization of *N*-aryl β-nitroenamines 20 in the presence of a tetrakistriphenylphosphine palladium(0) catalyst (Pd(PPh_3_)_4_) in good yield (eqn (8)). Although the described methodology was effective in introducing various electron withdrawing groups, strong withdrawing group, such as CN, NO_2_ and COOMe, caused a significant decrease of yield in indole formation.8
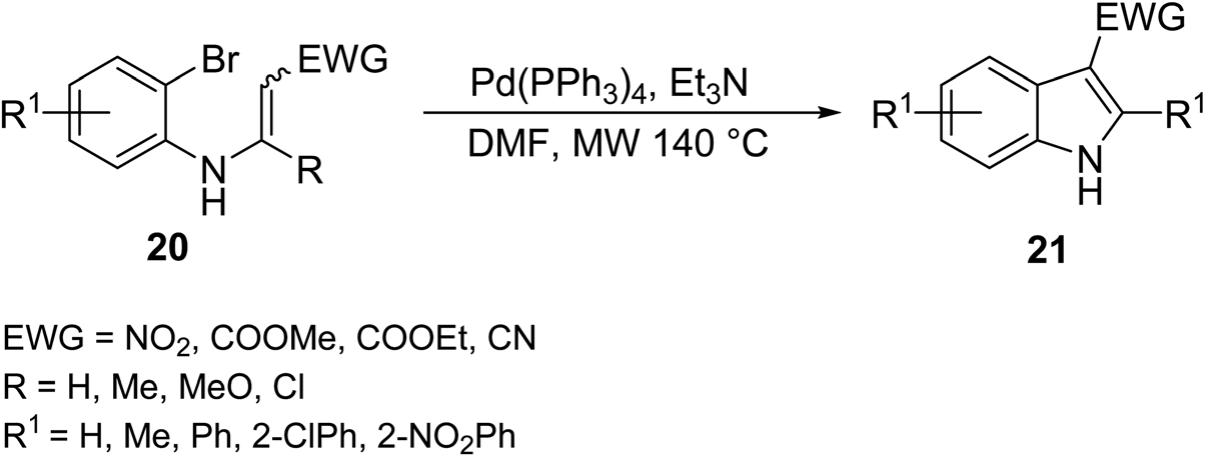


In an attempt to clear some hurdles encountered in obtaining indoles bearing electron withdrawing substrates, Carpita *et. al.*^26^ synthesized a series of 2,3-disubstituted indoles 23 and three azaindole derivatives bearing both electron-rich and electron-poor substrates and varied aromatic substituents *via* MW-assisted cycloisomerization of 2-alkynylanilines/pyrimidines 22 using water as a solvent and a basic/acidic salt as a catalyst (eqn (9)). Water is an attractive solvent alternative in green chemistry, but its main drawback is its inability to dissolve organic compounds; however, the superheating of water under MW irradiation gives it “pseudo organic” properties (by changing its dielectric profile), which enhances its solubility and efficiency in the reaction. The following methodology allowed for various functionalities to be appended on the indole architecture in good to excellent yields (60–99%) using water as a solvent.9
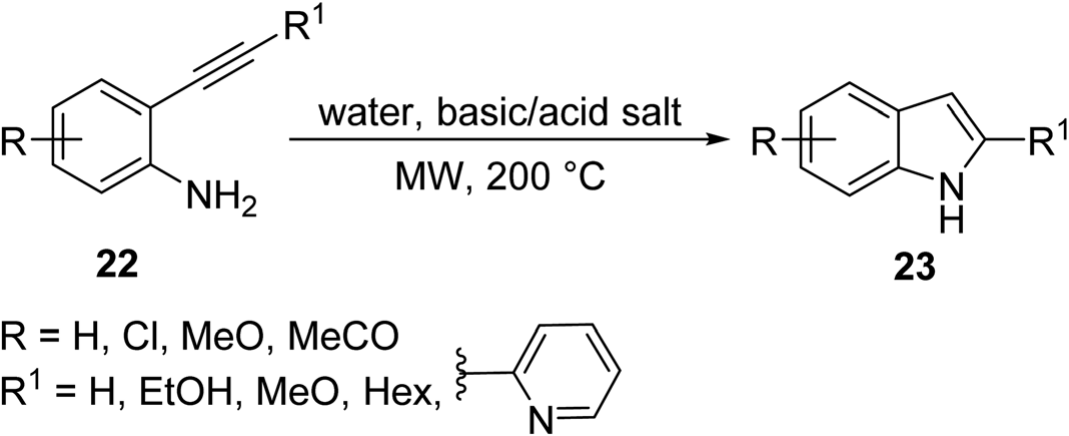


Higher order substituted indoles 26 were synthesized by Creencia *et. al.*^35^ through solvent-free, MW-assisted Fischer indole reactions of various substituted phenylhydrazines 24 and ketones 25 in the presence of a *p*-tolusulfonic acid catalyst (eqn (10)). The use of MW irradiation eliminated the need for solvents since *p*-TSA acted as both catalyst and solvent by directly transferring the heat to the reagent, leading to a cleaner reaction and easier workup. The reaction was performed in one step without the isolation of unstable arylhydrazones and resulted in higher yields and shorter reaction times compared to the non-MW assisted methods, which generally result in complex mixtures.10
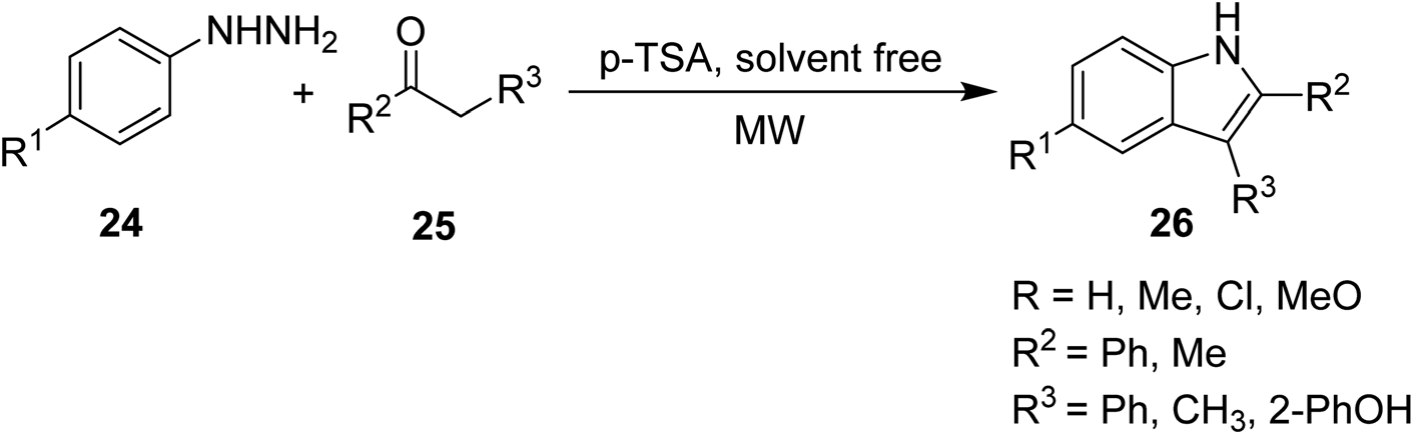


It is common in organic synthesis to combine multiple protocols in an effort to improve the efficiency of a reaction. The robust and diverse Fischer indole reaction was carried out by Porcheddu and his co-workers conjointly with an innovative hydrogen auto transfer technology, under MW irradiation, to catalytically oxidize readily available primary or secondary alcohols 28 to their respective carbonyl intermediates *in situ*. These then reacted with phenylhydrazine 27 in the presence of a protic molecule or Lewis acid to give the corresponding substituted indoles 29 (eqn (11)). The following methodology takes advantage of emerging technologies (hydrogen auto transfer and MW irradiation) to allow for the efficient conversion of readily available alcohols, in a Fisher fashion, into skeletally diverse indole nuclei under benign conditions.11
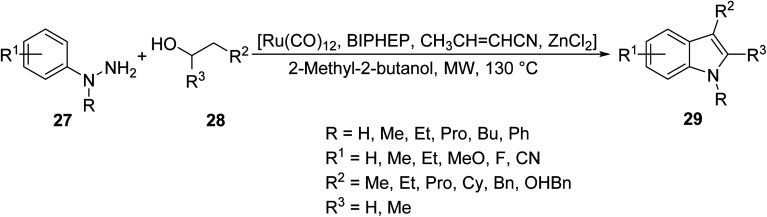


**Scheme 1:**
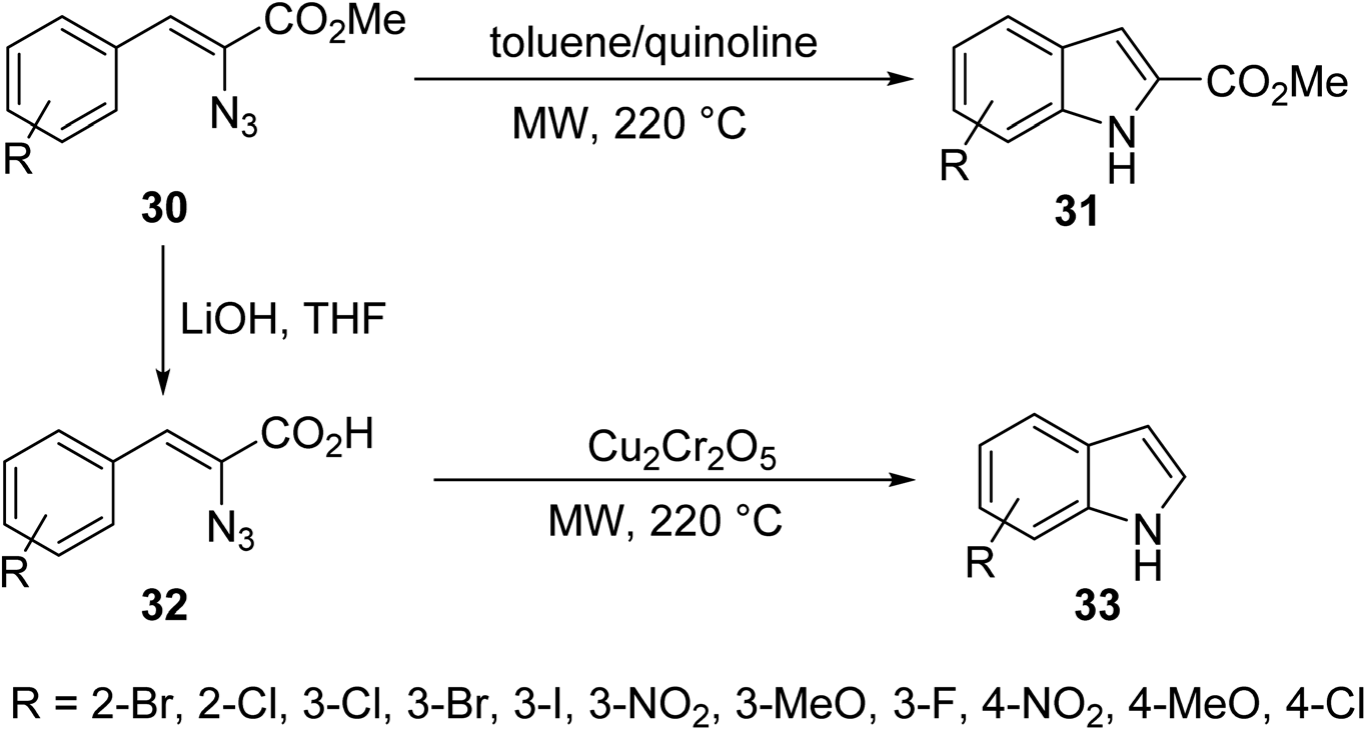
Synthesis of benzoindole derivatives by azidoester HK reactions.^36^

Ranasinghe *et. al.*^36^ described the design of a MW-assisted Hemetsberger–Knittel (HK) reaction of a series of azidoesters under a variety of conditions to produce a number of substituted indoles (Scheme 1). The azidoesters 30, readily obtained in one step, were directly cyclized into carboxylate indoles 31, or converted to their corresponding azidoesters 32. In the presence of a catalyst, monosubstituted indoles 33 were produced.

Conventional *versus* microwave methods for the synthesis of products 31 were explored and are highlighted in Table 2. Three variations of compound 31 were used as representative examples. In all cases, the microwave reaction time is just one twelfth that of conventional methods and results in a yield up to 24% higher than that of conventional methods.^36^

**Table 2:** Conventional *versus* microwave methods for products 31^36^

Method	R (Product)	Conditions	Reaction time (min)	Yield (%)
Conventional	Br	Xylene, 140 °C	120	66
Microwave	Br	Toluene, 200 °C	10	90
Conventional	NO_2_	Xylene, 140 °C	120	78
Microwave	NO_2_	Toluene, 200 °C	10	86
Conventional	OMe	Xylene, 140 °C	120	72
Microwave	OMe	Toluene, 200 °C	10	96

### Medicinal applications

**Fig. 4:**
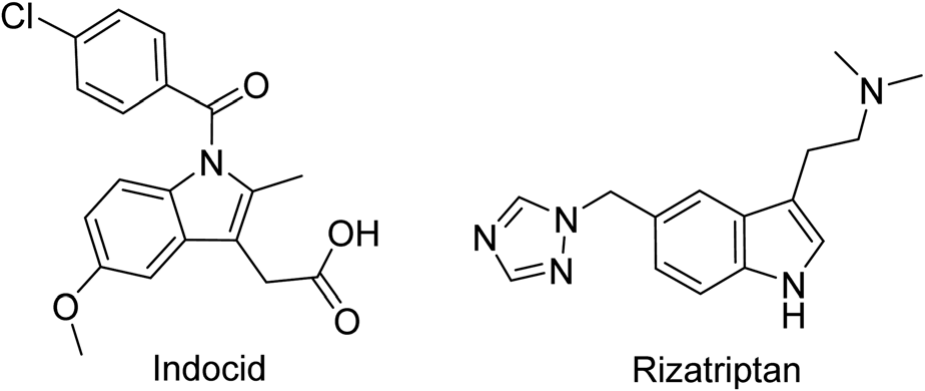
Drugs bearing the indole moiety.

The indole heterocycle shows several biological applications like anti-cancer, anti-bacterial, anti-viral, anti-inflammatory and anti-migraine activities.^37^ It is also used as an anti-depressant, anti-cholinergic, anti-emetic and anti-hypertensive. A lot of compounds are already on the market, like the non-steroidal inflammatory drug Indocid (Indometacina), that act as non-selective inhibitors of cyclooxygenase (COX-1 and COX-2) to treat chronic conditions such as rheumatoid arthritis, ankylosing spondylitis and osteoarthritis. By substituting the second, third, fifth and sixth positions of the ring, it is also possible to modulate antiviral activity. For instance, Enfuvirtide (T-20; Fuzeon), approved by the U.S. FDA in 2003, was the first HIV fusion/entry inhibitor for the treatment of HIV/AIDS. Indoles can also exhibit strong anticancer activities as is shown in drugs such as Sunitinib, used for renal cell carcinoma, Osimertinib, used for the treatment of gastrointestinal stromal tumor and NSCLC adenocarcinoma, and Alectinib, an ALK inhibitor implicated for Crizotinib resistant NSCLC.^38^ Recently, 2-carboxyindole derivatives were synthesized by Cury *et. al.* and tested for their activity against acute lymphoblastic leukemia. The derivatives showed selectivity and promising effectivity toward leukemia cells.^39^ In addition, indoles have a wide range of applications including antimicrobial, antiemetic and migraine activities, among which “Triptans” were approved between 1992 and 2001 and contain an indole ring as their basic scaffold (Fig. 4).^40^

## 4. Pyridine

In response to the need for developing environmentally friendly methods for the synthesis of pyridine compounds starting from cheap and readily available reagents, Bayramoǧlu *et. al.*^41^ investigated a MW-assisted conversion of glycerol 34, a by-product in the production of biodiesel, in the presence of ammonium salts and acid catalyst, to 3-methylpyridine 35 and pyridine 36 (eqn (12)). The reaction proceeds through the *in situ* dehydration of the glycerol to form acrolein (as well as small carbonyl compounds), which sequentially reacts to give the product.12
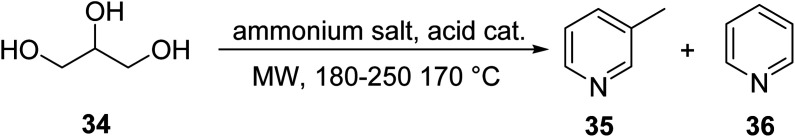


**Scheme 2:**
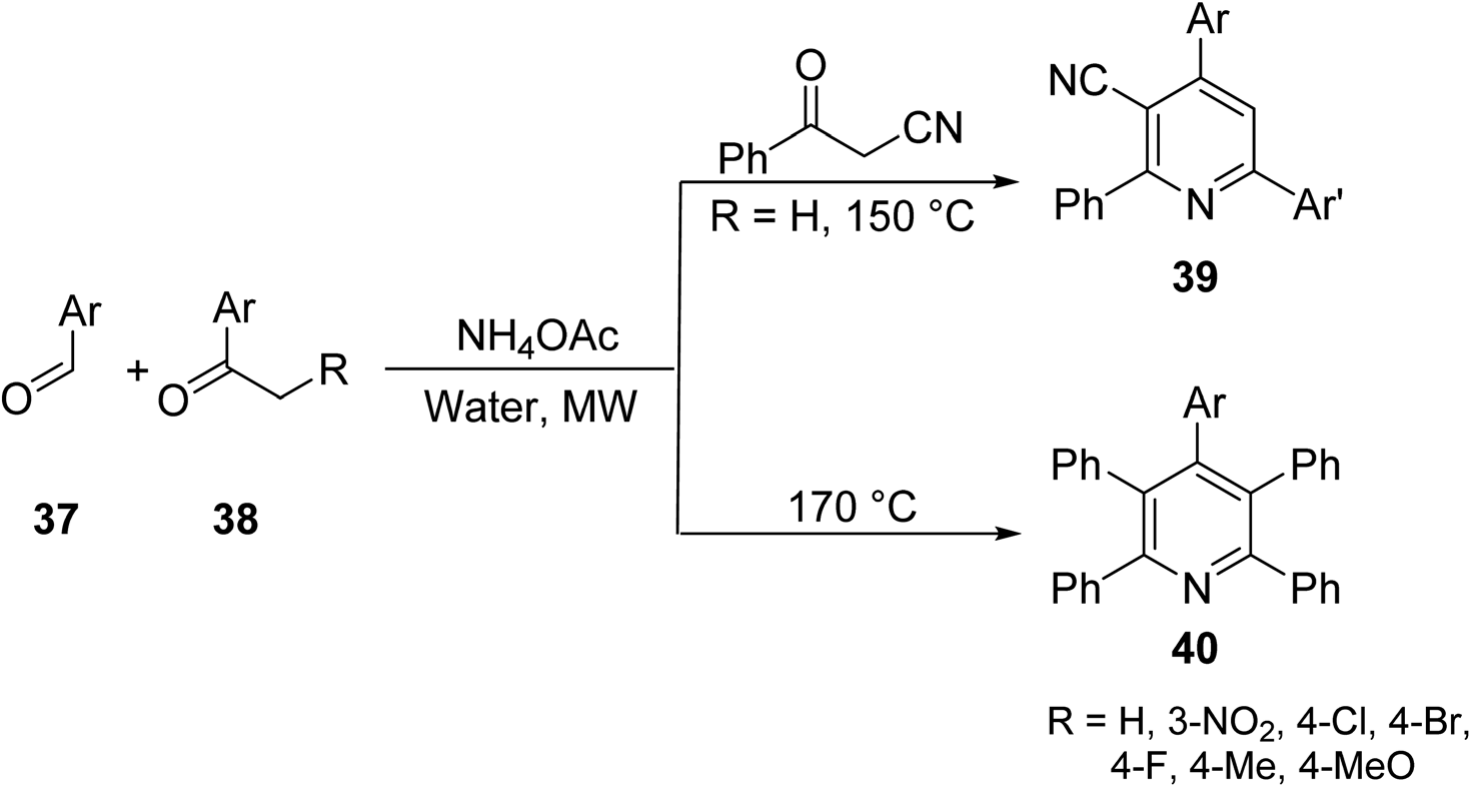
Synthesis of 2,4,6-triarylpyridines *via* aromatic aldehydes and 2-acetylaromatic compounds.^42^

Jiang *et. al.*^42^ explored a new and improved protocol to form highly functionalized 2-pyridines (Kröhnke pyridines). This methodology offers a simple and efficient route for the diversity-oriented synthesis, DOS, of pyridine derivatives using MW irradiation. The goal of DOS is the facile preparation of collections of structurally complex and diverse compounds from simple starting materials. Various aromatic aldehydes 37 and 2-acetylaromatic substrates 38 were reacted, in the presence of ammonium acetate, to yield 3-oxo-3-phenylpropanenitrile for unsymmetrical 2,4,6-triarylpyridines 39 or penta-aryl pyridines 40 (Scheme 2). The DOS methodology under MW heating provides rapid access to pyridines with predictable control to selectively introduce various substituents.

In a continuous effort to develop facile routes for useful, functionalized heterocycles, Jiang introduced a new four-component domino reaction for the synthesis of polyfunctionalized pyridine derivatives.^43^ The reaction was achieved by reacting malononitrile 42 and various cycloketones 41 (2 eq. of each) with ammonium acetate in a one-pot reaction under MW irradiation (eqn (13)).13
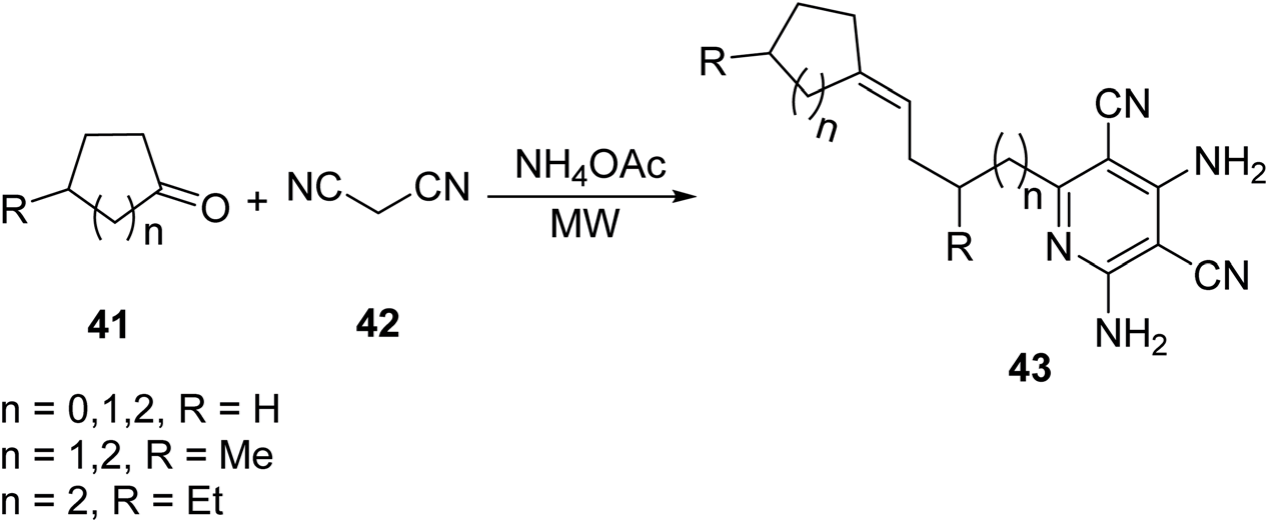


Annulated pyridines, a subclass of pyridines bearing fused cyclic/aryl moieties, constitute an important class of biologically active compounds that have been explored for an array of use in medicinal chemistry including antimalarial and antitumor activites. The increase in the application of this subclass of heterocycles has made it important for medicinal chemists to understand their preparation and develop methods to increase the efficiency of their synthesis. In this context, Vasilyev^44^ developed a one-pot metal catalyzed reaction of pinocarvone oxime 44 with enamines 45 to afford substituted annulated pyridines bearing chiral nopinane 46 (eqn (14)). Chiral pyridines are used in asymmetric synthesis as catalysts, but most reported routes for their synthesis involve multiple steps. The following MW-assisted methodology produces a variety of pinane–pyridine hybrids in fewer steps and with shorter reaction times.14
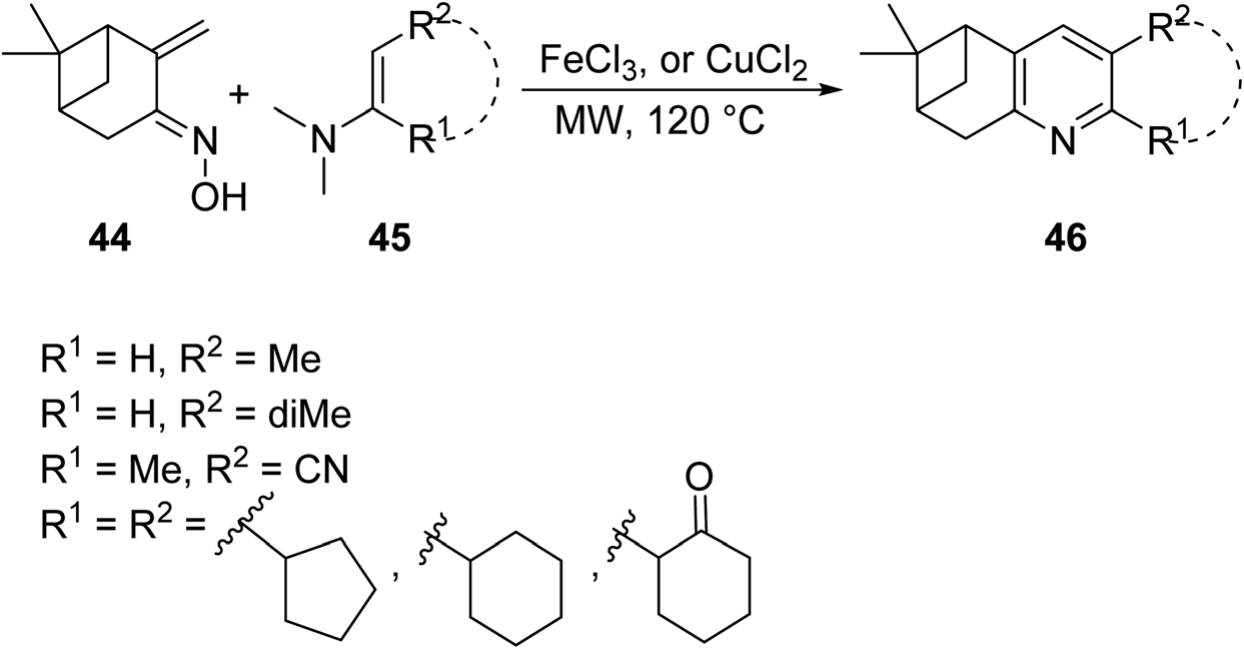


Shekarrao *et. al.*^45^ developed a ligand-free, copper-catalyzed, three-component synthesis for a variety of annulated pyridines 50 (and benzonitrile 51 as secondary product) through the reaction of β-halovinyl/aryl aldehyde 47, aromatic/aliphatic terminal alkyne 48 and *tert*-butylamine/benzamidine 49 in DMF under MW irradiation (eqn (15)). Regioselectively annulated pyridines constitute an important scaffold of biologically active compounds that have found use as anticancer therapeutics.15
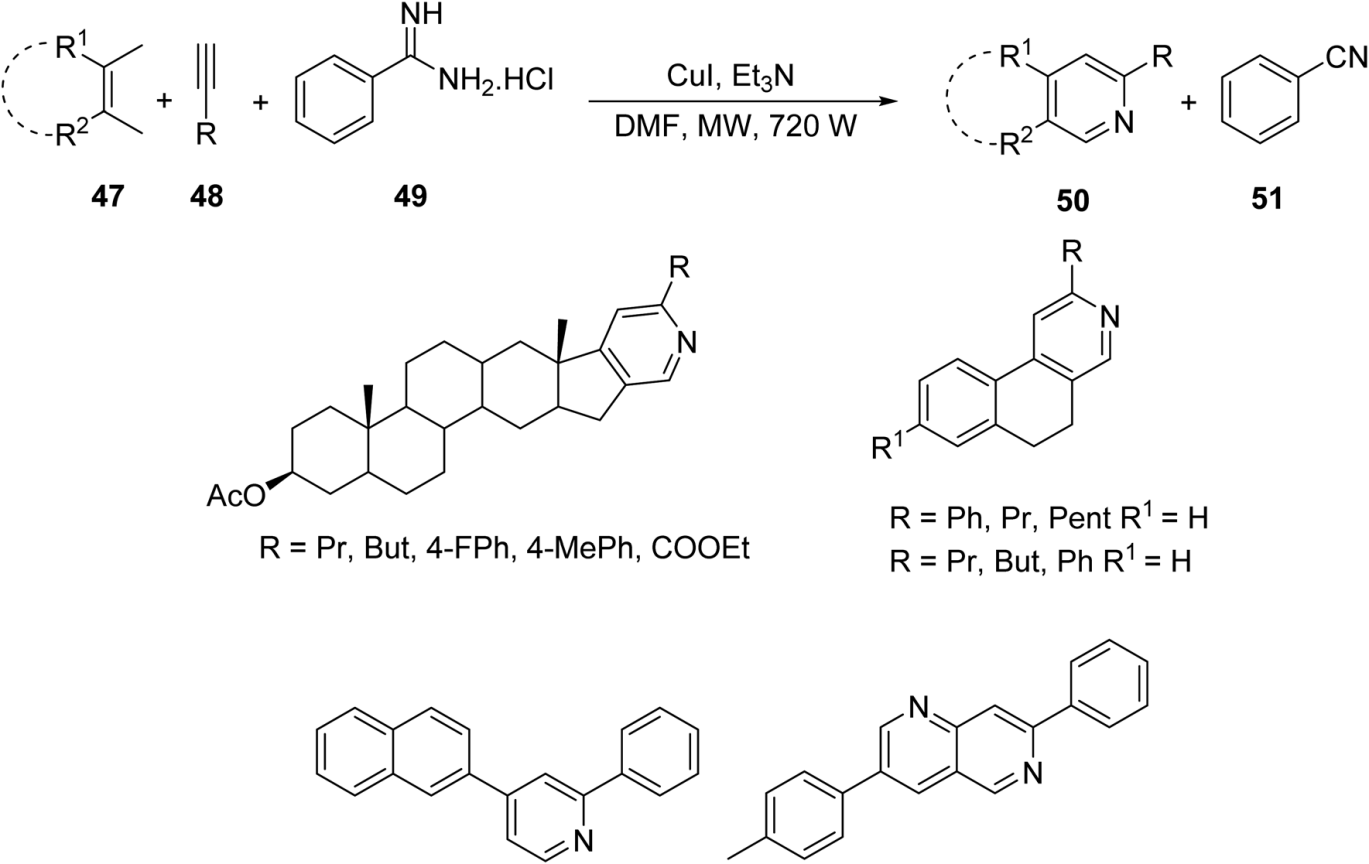


Lee *et. al.*^46^ investigated a novel MW-promoted, one-pot reaction of aryl and α,β-unsaturated ketones 52, alkynes 53 and ammonium acetate 54 to produce N-annulated pyridine derivatives 55 bearing diverse cyclic substituents (eqn (16)). This protocol allows for the synthesis of “crowded” pyridines in high yields, and thus bypasses steric effects, which typically decrease the efficiency of their synthesis.16
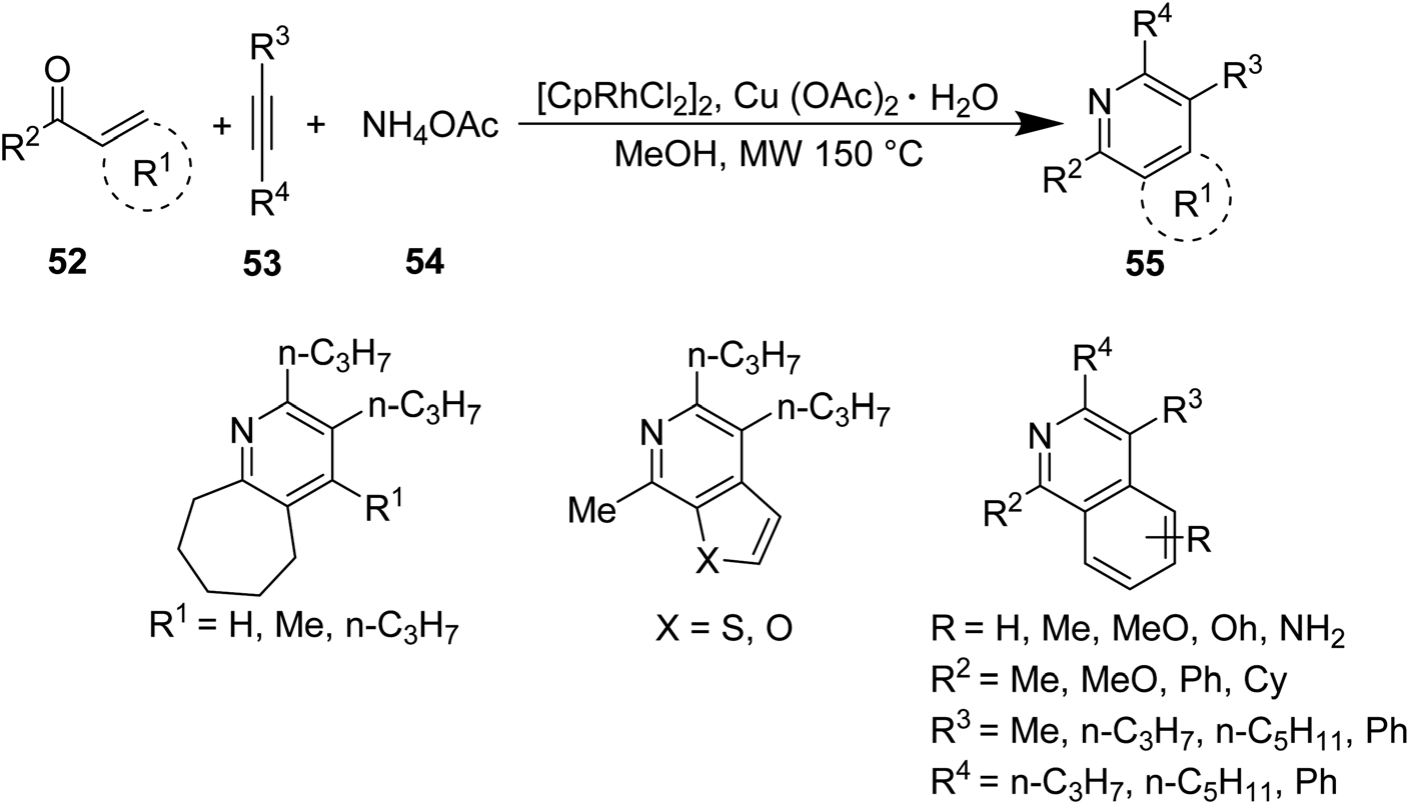


**Scheme 3:**
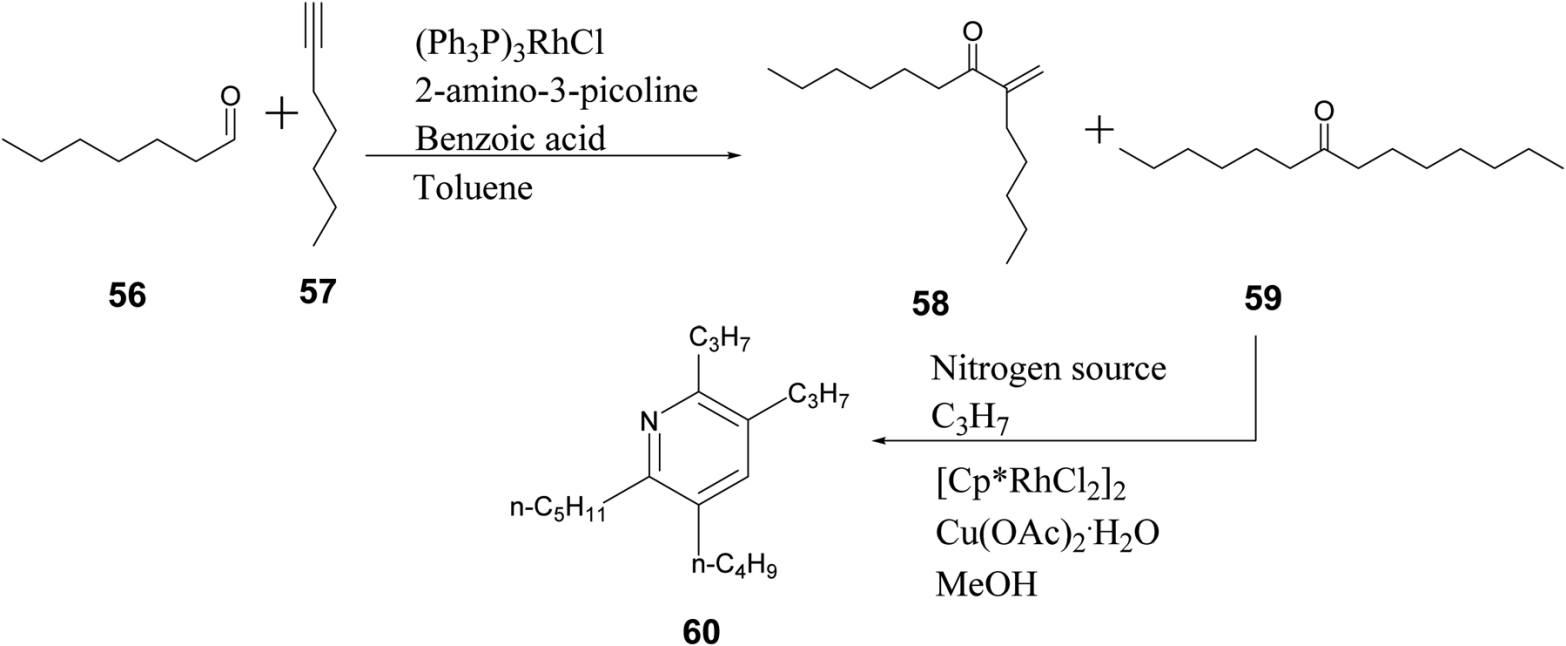
Multi-step synthesis of pyridine derivatives.^47^

Similar compounds were synthesized by Sim *et. al.* through conventional methods. Compound 60, synthesized through conventional methods, is depicted in Scheme 3. It was compared with compound 55 containing R_1_ = benzyl, R_2_ = *n*-C_5_H_11_, R_3_ = *n*-C_3_H_7_, and R_4_ = *n*-C_3_H_7_.^47^

The differences between conventional and microwave synthesis of these products is vastly different. One example of each compound is used as a representative example. Microwave reactions occur in 1.67% the time of that with conventional methods and result in the same yield (Table 3).

**Table 3:** Comparison of reaction conditions for substituted pyridine compounds^46,47^

Compound	Method	Reaction time (min)	Yield (%)
60	Conventional	600	92
55 (R_1_ = benzyl, R_2_ = *n*-C_5_H_11_, R_3_ = *n*-C_3_H_7_, and R_4_ = *n*-C_3_H_7_)	Microwave, 150 °C	10	92

### Medicinal applications

**Fig. 5:**
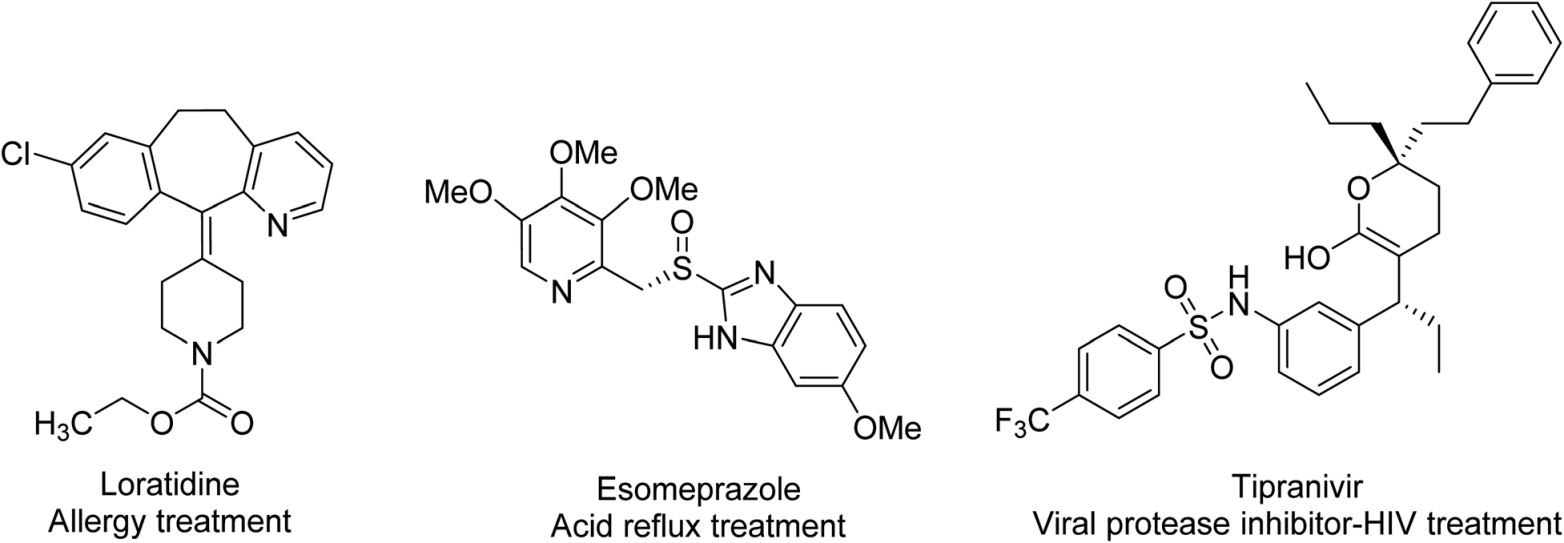
Drugs that exhibit a pyridine structure.

Pyridine-based chemical compounds rank among the most highly represented in the pharmaceutical industry, with many having several key biological characteristics. Examples include pyrimidine/hexahydroquinazoline-fused pyrazolo[3,4-*b*] pyridine derivatives designed and synthesized by Pradeep that show antibacterial, antifungal and anti-biofilm activities.^48^ Pyridine derivatives like 2-acetyl and 4-acetyl pyridines condensed with amides also have excellent anti-inflammatory activities as well as anti-cancer, anti-diabetic, antioxidant, anti-viral, analgesic and anti-malarial properties.^49^ Moreover, drugs with pyridine are used in clinical practice in the treatment of rheumatoid arthritis, ulcerative colitis and Crohn's disease, but they have to be conjugated to sulfapyridine and 5-aminosalicylic acid (the compound called sulfasalazine) or be administered as a prodrug to avoid crystallization in the bladder and urethra.^50^ Pyridine derivatives can also exhibit selective antibacterial activity against Gram negative bacteria *E. coli* and Gram positive bacteria *S. albus*. They have also been tested as herbicides against *C. dactylon*, *C. rotundus*, *E. crusgalli*, *E. hirta*, *C. argentia*, *E. indica* and *T. procumbens*.^51^ Recently, isothiazolo[4,3-*b*]pyridines were synthesized and tested as cyclin G-associated kinase inhibitors and for anti-viral activity. With changes in pyridine substitution, these compounds were found to show promising activity toward dengue virus in particular.^52^Fig. 5 shows three important compounds on the market that utilize pyridine; among them are Esomeprazole (Nexium™), which ranks 2^nd^ in U.S. pharmaceutical market sales.

## 5. Pyrrolidine

MW technology has led to the improvement of some synthetic routes in the creation of novel pyrrolidine scaffolds. Perhaps, with the expansion of MW-assisted chemistry towards the optimization of innovative methods for the synthesis of this heterocycle, we may see more bioactive compounds. Chang synthesized a series of N-substituted pyrrolidine derivatives 62*via* amination reactions of dimesylate 61 with various alkylamines in excellent yield (70–99%) using MW irradiation (eqn (17)).^53^ In the conventional synthetic route, low or no yield was obtained. Subsequent deprotection of the following pyrrolidines lead to the formation of their hydroxylated analogues, iminosugars, which have shown potency as glycosidase and glycosyltransferase inhibitors.17
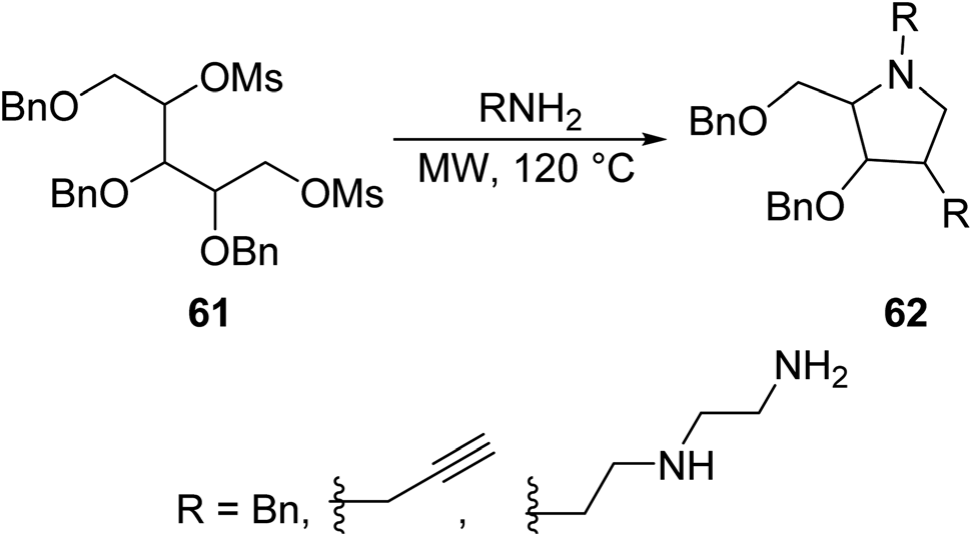


Li *et. al.*^54^ developed a greener approach to the synthesis of *N*-arylpyrrolidines 65 by reacting arylamines 63 with 1,4-dimesyloxybutane (1.3 eq.) 64 in water, in the presence of aqueous alkaline medium potassium carbonate (K_2_CO_3_) (eqn (18)). Activated aniline and *p*-toluidine were easily cyclocondensed in good yields, and those with strong deactivating groups were obtained in lower yields.18
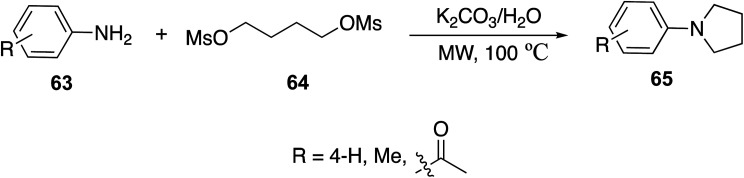


Ju *et. al.*^55^ synthesized *N*-arylpyrrolidine 68, a derivative of compound 65, through eqn (19) by conventional and microwave methods. This molecule was chosen as a representative example. The results are shown in Table 4.19
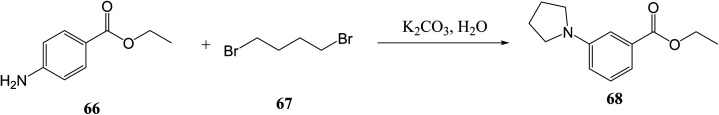


**Table 4:** Comparison of conventional and microwave methods in the synthesis of compound 68^55^

Method	Time (min)	Yield (%)
Conventional	480	58
Microwave (120 °C, 80–100 W)	20	93

### Medicinal applications

**Fig. 6:**
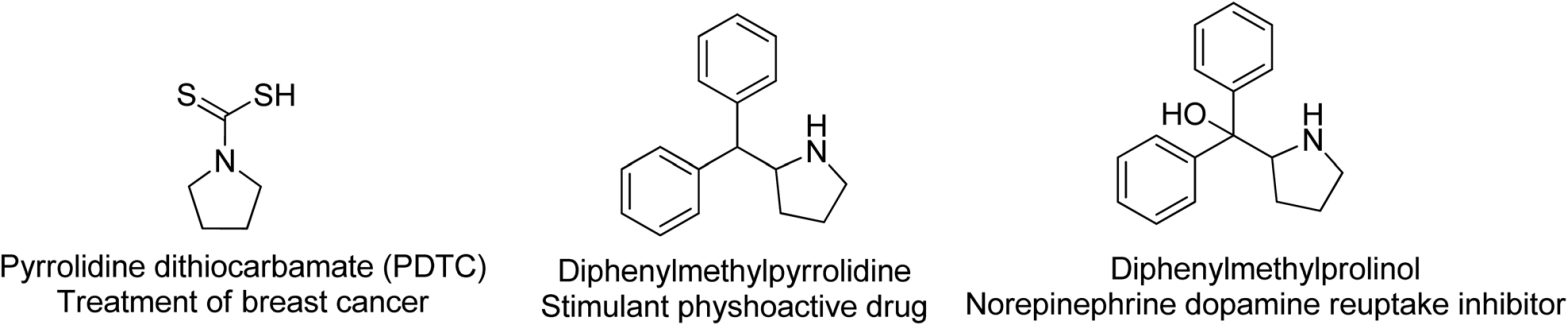
Examples of pyrrolidine based pharmaceutical agents.

Pyrrolidines are strongly basic secondary amines that can be found in many natural products; however, their widespread utility in medicine has been niched into the psychoactive market, with several reuptake inhibitors utilizing this heterocycle. Also, the dithiocarbamate analog (Fig. 6) showed some promise in the treatment of breast cancer. Dimethylamino pyrrolidines have been recently reported as novel inhibitors of the PRC2 complex through the disruption of EED/H3K27me3 binding.^56^ Recently, the application of pyrrolidines as spiro-compounds has been investigated. For instance, it has been discovered that molecules containing a spiro-[indole-pyrrolidine] moiety are cell-cycle-specific cytostatic agents that arrest mitosis and metaphase. They are also found to be useful in cancer chemotherapy against the proliferation of MCF-7 breast cancer cells^57^ or against MCF-7 breast cancer cell line by the MTT assay.^58^ They can also exhibit antimycobacterial (*Mycobacterium tuberculosis* H37Rv (MTB), multi-drug-resistant *Mycobacterium tuberculosis* (MDR-TB) and *Mycobacterium smegmatis*) and antifungal activities. Furthermore, many spiro-compounds containing pyrrolidine have been lately used as privileged scaffolds for the development of therapeutic molecules such as anticonvulsant, antituberculosis, anti-Alzheimer's, pain-relief and antidermatitis agents. For example, Saraswata developed spiropyrrolidines and oxindole moiety derivatives applied to microbial infections related to viral and HIV infections.^59^ Recently, polyhydroxylated pyrrolidine derivatives were developed by Guazzelli *et. al.* and shown to inhibit alpha-glucosidase and aldose reductase, which are both factors in diabetes.^60^

## 6. Imidazole

Polyaromatic imidazoles are generally synthesized through the Debus-Radziszewski imidazole condensation of 1,2-dicarbonyl, aldehydes and ammonium acetate or a variation thereof. Chundawat^61^ incorporated MW protocols into the Debus-Radziszewski imidazole. Triphenyl imidazole derivatives 71 were obtained by reacting substituted benzaldehyde 70 with benzil 69 and ammonium acetate in the presence of a Schiff base nickel complex (Ni–C) catalyst. The following condensation facilitated the synthesis of a series of phenyl-substituted imidazole rings shown as templates for the rapid, MW-assisted functionalization for the development of potent, biologically active imidazoles (eqn (20)).20
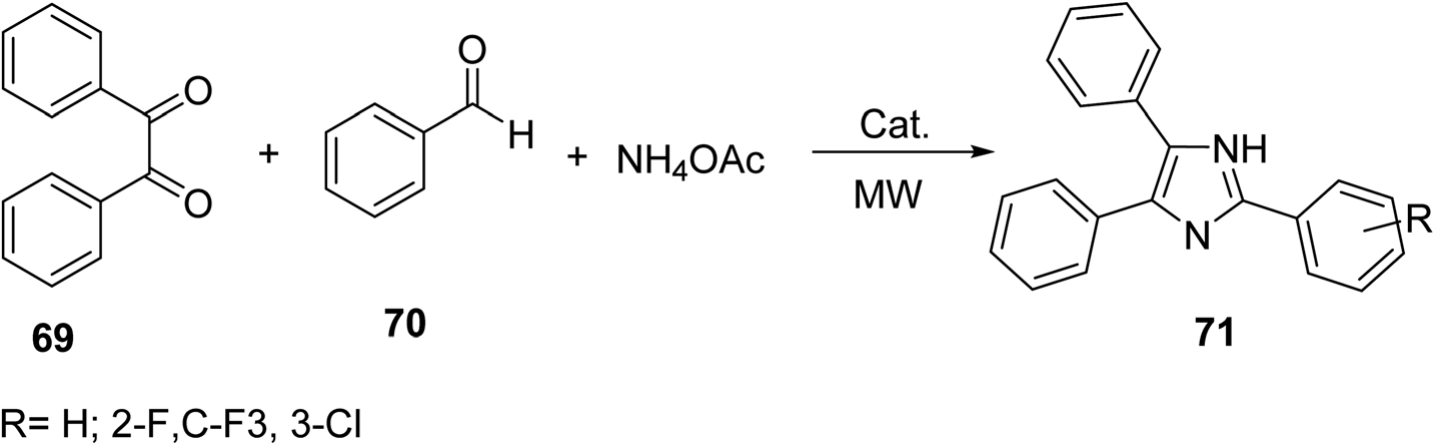


Products 71 have been synthesized by conventional and microwave methods. Four variations of compound 71 were used as representative examples. Microwave methods show a drastic decrease in reaction time and increase in product yield, highlighting its advantage in the synthesis of imidazole derivatives (Table 5).

**Table 5:** Comparison between conventional and MW protocols in the synthesis of products 71

R	Conventional (reflux)		MW	
	Reaction time (min)	Yield (%)	Reaction time (min)	Yield (%)
H	270	70	20	90
3-Cl	300	68	25	86
4-MeO	240	72	15	88
3,4-MeO	240	70	15	85

**Scheme 4:**
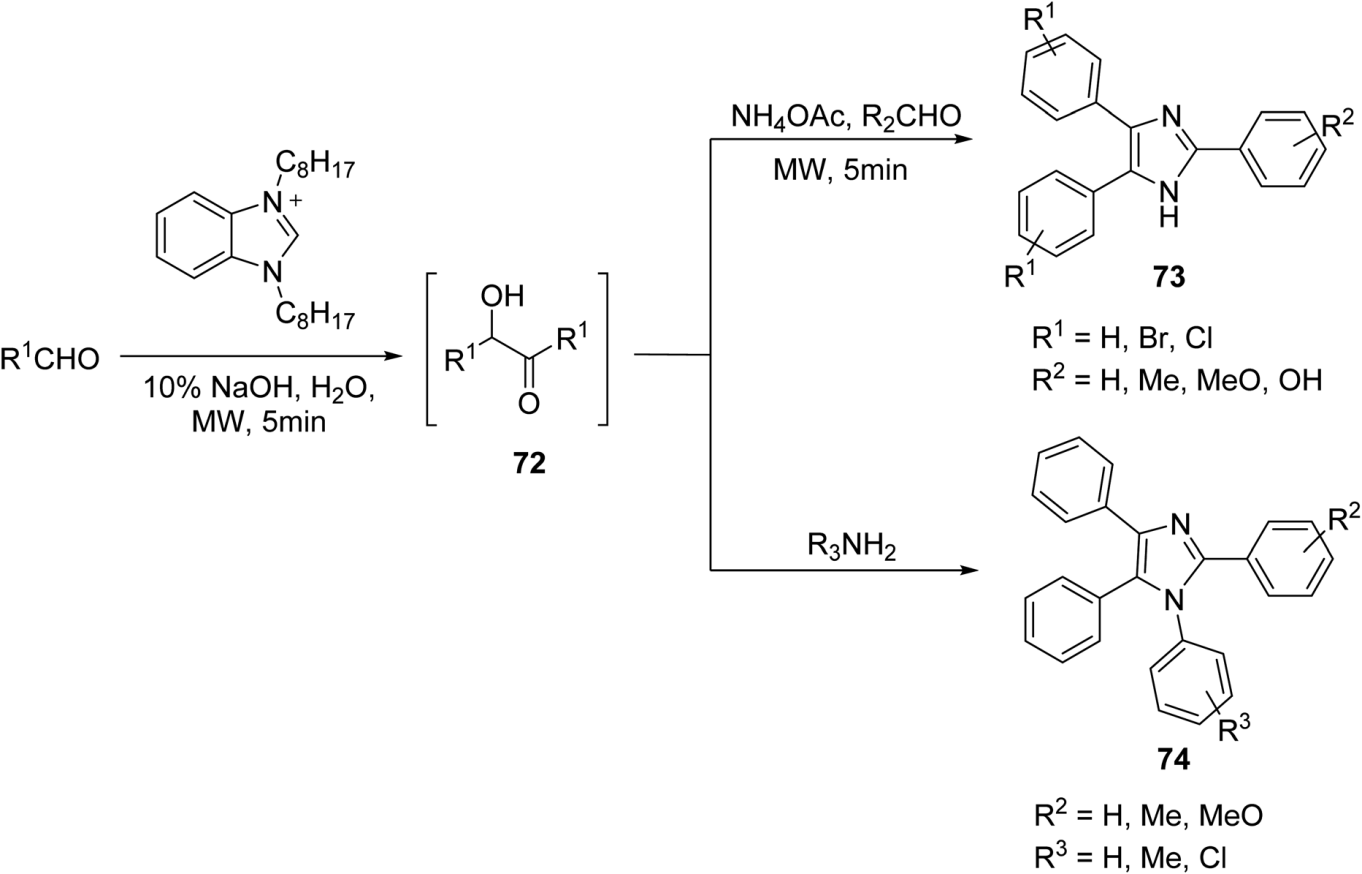
Synthesis of tri- and tetra-substituted imidazole analogs *via* arylaldehydes.^62^

In order to diversify the imidazole center, the synthesis of polyaromatic imidazoles bearing different substituents on aryl groups is introduced by Wu, starting from arylaldehydes that were converted *in situ* to their respective benzoin intermediate 72 and reacted with ammonium acetate and arylaldehyde or arylamine under MW irradiation to yield imidazole derivatives 73 and 74, respectively (Scheme 4).^62^ The following methodology afforded trisubstituted imidazoles bearing chloro, bromo or methyl groups on the phenyl ring in good yields, while their furanyl, pyridinyl, nitro and hydroxyl phenyl correspondences could not be obtained.

Mirjafari *et. al.* was able to address the diversification of the imidazole nucleus by introducing diverse moieties including furanyl, pyridinyl, nitro and hydroxyl phenyl to its scaffold through a MW-mediated reaction of α-hydroxyketones 72, heterocyclic alcohols 75 (instead of aldehydes) and ammonium acetate in the presence of ionic liquid 1-methyl-3-H -imidazolium nitrate (eqn (21)).^63^21
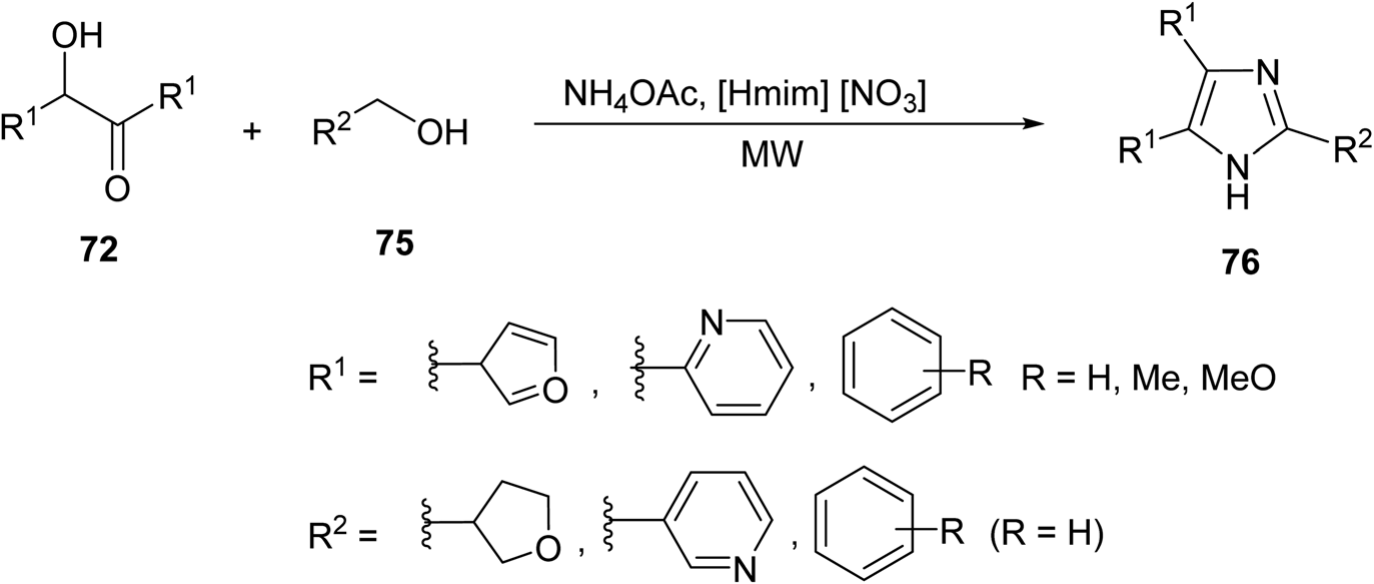


**Scheme 5:**
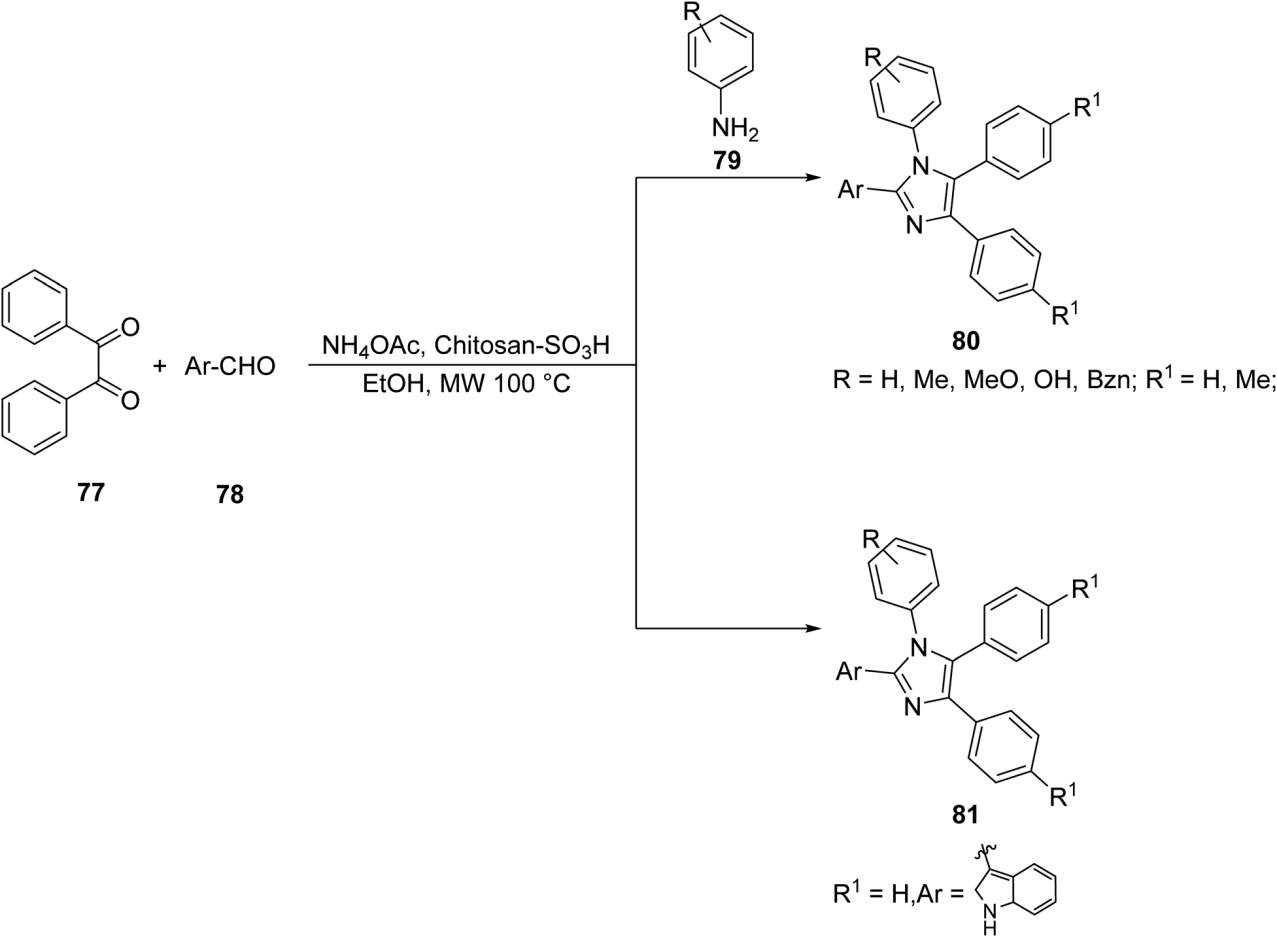
Synthesis of polycyclic imidazole derivatives *via* benzyl.^64^

Khan *et. al.*^64^ utilized a multicomponent condensation of polycyclic heterocycles jointly with green chemistry to develop a powerful pathway to synthesize tri- and tetra-substituted imidazoles. Benzyl 77 was reacted with various aromatic aldehydes 78 with ammonium acetate or substituted aniline 79 under MW heating, using functionalized chitosan as a biodegradable solid acid catalyst to yield imidazoles 80 and 81, respectively (Scheme 5). Chitosan is a heterogeneous biopolymer-supported solid acid catalyst that can absorb MWs and directly heat the reaction mixture. This proved to be an advantageous method in the green creation of polycyclic imidazoles in terms of yield (85–96%) and efficiency.

The syntheses of compounds 80 and 81 through conventional and microwave methods were compared and are shown in Table 6. Two variations each of compounds 80 and 81 were chosen as representative examples. In every case, the reaction time for microwave synthesis is a fraction of that with conventional methods and the yield is the same or better.

**Table 6:** Comparison of conventional *versus* microwave methods in the synthesis of substituted imidazoles. Microwave irradiation was conducted at 5–10 W and 100 °C^64^

Compound	R	R_1_	Reaction time (min)		Yield (%)	
			Conventional	Microwave	Conventional	Microwave
80a	H	H	420	90	87	91
80b	OCH_3_	H	360	72	87	89
81a	H	H	630	120	90	90
81b	OCH_3_	H	420	90	88	89

### Medicinal applications

**Fig. 7:**
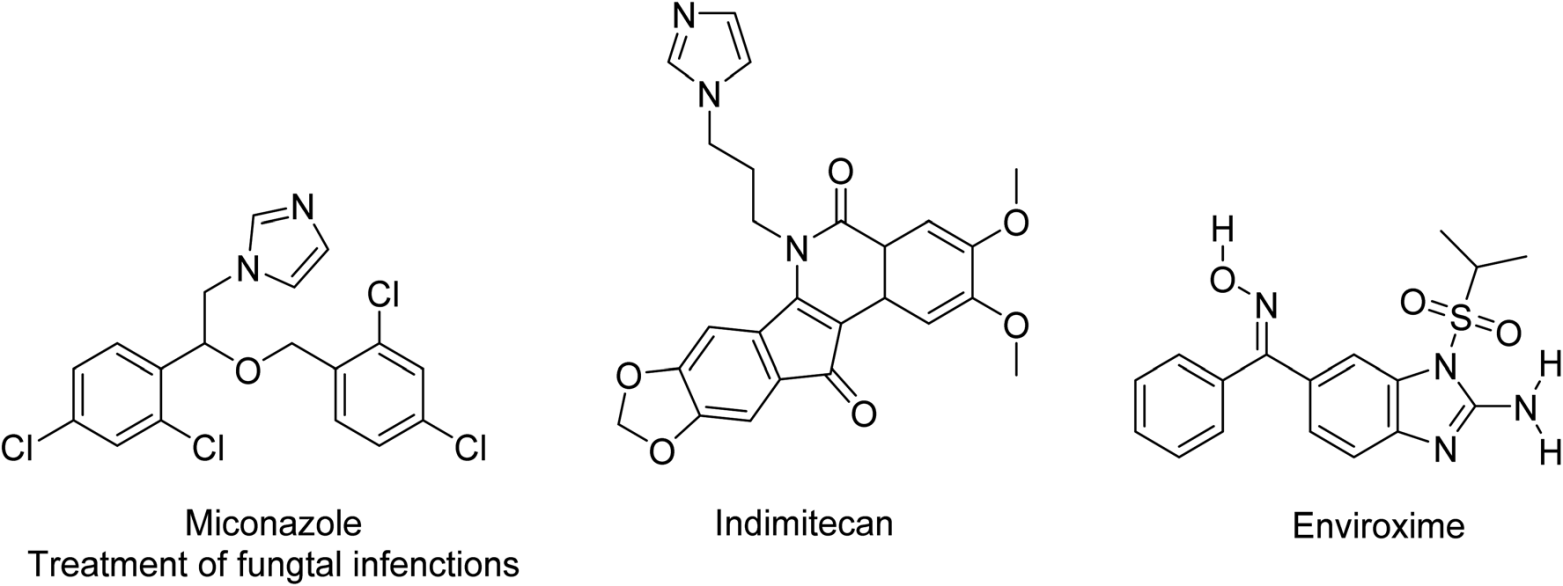
Selected drugs containing imidazole ring for fungal infections.

MW methods for the preparation of functionalized imidazole rings are highly desirable, especially in the combinatorial synthesis of new antifungal agents. Many different medicinally relevant agents rely on an imidazole ring for their biological activities. As shown in Fig. 7, imidazoles are found as active heterocyclic centers in the treatment of many fungal infections, but also as curing agents for epoxy resins or in agrochemicals. Ramanathan described the antimicrobial activity of 2-(4-methoxynaphthalen-1-yl)-1-(4-methoxyphenyl)-1*H*-phenanthro[9,10-*d*]imidazole against bacterial strains of *S. aureus*, *S. typhi* and *E. coli*.^65^ The same molecule also exhibited moderate to maximum antifungal activity against *Candida albicans, Aspergillus niger* and *Mucor*.^65^ Imidazole-based drugs have been approved for clinical use as anticancer drugs – indimitecan has been demonstrated to inhibit the topoisomerase-I enzyme by intercalating between DNA base pairs to stabilize a ternary complex.^66^ Benzimidazole has several biological applications and can be viewed as an extended imidazole scaffold as well as an indole bioisostere.^67^ Beck describes how it can result in a different spectrum of anticancer activities due to the production of various DNA cleavage sites relative to camptothecins, and therefore targets genes differently.^68^ Benzimidazole rings may be responsible for the purine analog activity of bendamustine, as described by Weide *et. al.* Recently, Das *et. al.* repurposed imidazole derivatives to combat Newcastle disease virus, which has caused a global epidemic.^69^ This heterocycle can also have medicinal activity as an anticonvulsant agent, anti-virus agent (like Enviroxime and related compounds), antithyroid agent, antidiabetic agent, sedative agent, hypnotic agent (such as Zolpidem), anesthetic, immune suppressant, anticoagulant, blocker of retinoic acid metabolism, thromboxane synthase inhibitor and analgesic.

## 7. Pyrazole

In the quest to synthesize a relevant pyrazole scaffold, Vaddula^70^ introduced a solvent-free MW-assisted synthesis of a tetra-substituted pyrazole *via* the reaction of aryl hydrazines 82 and 3-substituted-pentane-2,4-diones 83 (eqn (22)). MW heating allowed the efficient synthesis of various aryl substituted pyrazole in high yield without the need for further purification. The reaction protocol could also be extended for the synthesis of diazepines.22
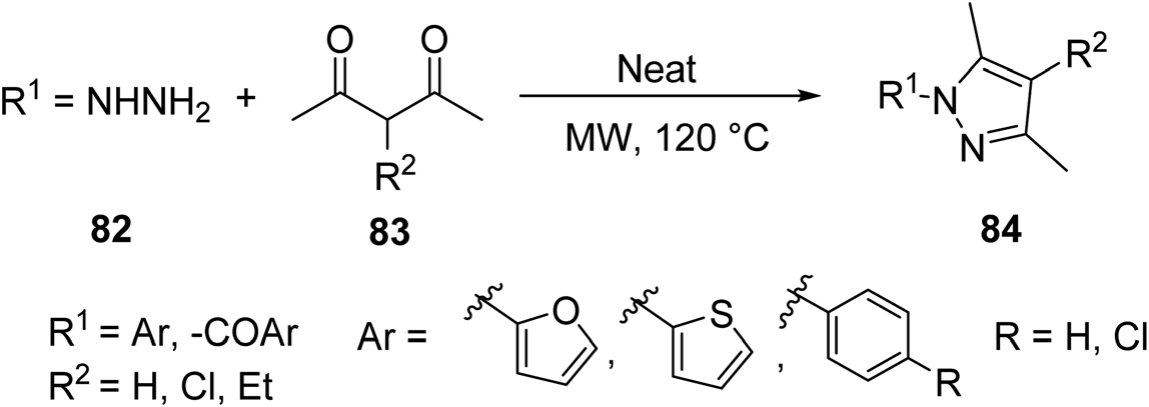


Du *et. al.*^71^ incorportated the above-mentioned protocol into the synthesis of some novel sugar-based pyrazole derivatives 87 through the MW-assisted cyclization of sugar-based phenyl hydrazide 85 with corresponding 2,4-pentanedione 86 in water (eqn (23)). Sugar-based pyrazoles have shown promising antitumor activity.23
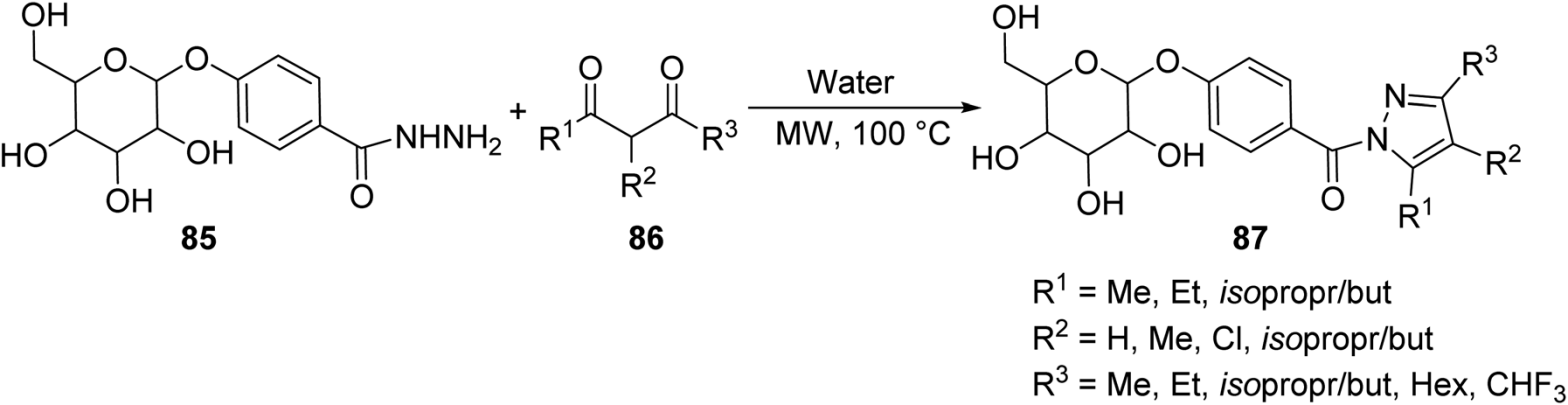


Compound 87, in which R_1_ = Me, R_2_ = H, and R_3_ = Me, was synthesized through conventional and microwave methods. One derivative of compound 87 was chosen as a representative example. In this case, conventional methods result in no yield at all, deeming it useless. Clearly, microwave methods are superior due to their low reaction times and relatively high yields. These methods are shown in Table 7.

**Table 7:** Conventional *versus* microwave methods in the synthesis of sugar-based pyrazole derivative 87^71^

Method	Solvent	Reaction time (min)	Yield (%)
Conventional (reflux)	H_2_O	300	0
Microwave (500 W, 100 °C)	H_2_O	15	88

Le Corre *et. al.* synthesized substituted pyrazoles 89 bearing three different functionalities (nitrile, ester and amine) through a MW-assisted Thorpe–Ziegler cyclization of dicyanohydrazones 88 in the presence of methyl bromoacetate and potassium carbonate (eqn (24)).^72^ This class of joined aromatic/heteroaromatic pyrazole units represents an important scaffold in drug design resembling nucleic acid base pairs (cytosine and thymine), whose geometries and electronic distributions provide the basis of action for many known therapeutics.24
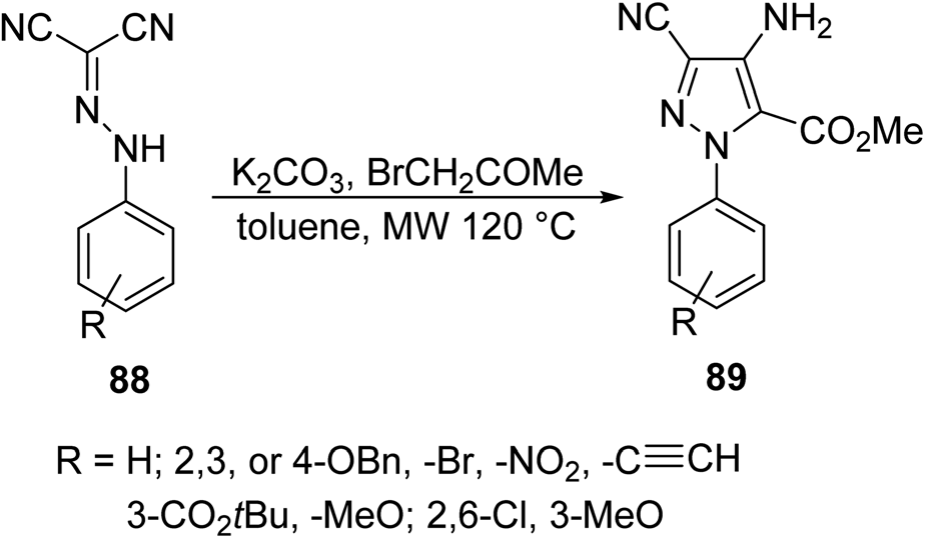


### Medicinal applications

**Fig. 8:**
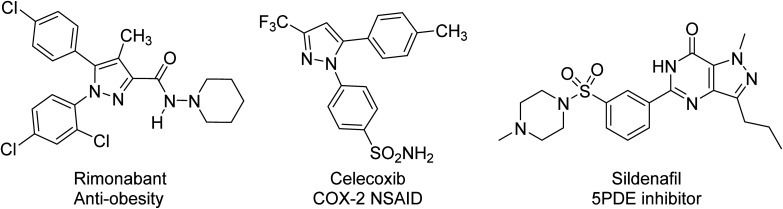
Pyrazole containing pharmaceutical agents.

Literature reports that derivatives of pyrazole have played a crucial role in the history of heterocyclic chemistry and have been used extensively as important pharmacophores. For example, Czarnomysy *et. al.* synthesized six novel complexes of platinum(ii) with pyrazole that exhibited cytotoxic activity against MCF-7 and MDA-MB-231 breast cancer cell lines.^73^ The cytotoxicity has been evaluated toward colon carcinoma HCT116 and leukemia K562 cultured human tumor cells.^74^ Other pyrazole drugs with biological activities are Rimonabant, which functions as a cannabinoid receptor and is utilized to treat obesity, Celecoxib, which inhibits COX-2, Fomepizole, an alcohol dehydrogenase inhibitor and Sildenafil, which selectively inhibits phosphodiesterase type 5.^75^ Recently, pyrazole derivatives with 5-phenyl-2-furan substitutions were synthesized by Ahmed *et. al.* and tested for their antifungal activity. Certain variations of this compound showed high antifungal activity, specifically toward *P. infestans*.^76^ Pyrazole has also been used as a privileged scaffold for monoamine oxidase inhibitors, protein B-Raf inhibitors, DNA gyrase inhibitors, anti-hepatotoxicity agents, antileishmanial agents, analgesics, cyclin-dependent kinase inhibitors, IRAK4 inhibitors, antibacterial and antifungal activities, antioxidants and 5α-reductase inhibitors (Fig. 8).^77^

## 8. Pyrazoline

The synthetic routes to new substituted pyrazolines have been investigated in recent years and novel pyrazolines are easily accessible from commercially available starting materials. As many pyrazolines are obtained from chalcone derivatives, Tripathi *et. al.*^78^ employed cyclization of various chalcones 90, obtained by aldol condensation of aromatic/heteroaromatic aldehydes and ketones, with hydrazine hydrate in the presence of a base using conventional and MW protocols to form 3,5-disubstituted pyrazoline derivatives 91 bearing a variety of aryl substituents (eqn (25)).25
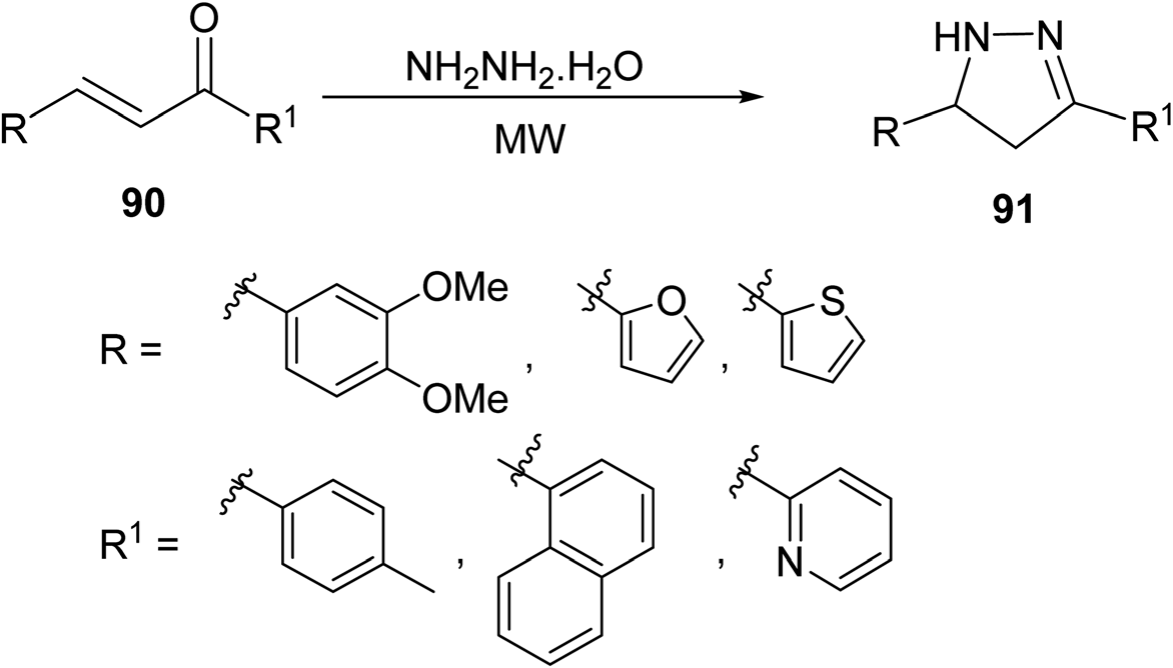


Saber *et. al.* continued to diversify the functionalities and procedures of acquiring novel pyrazoline systems by introducing a regioselective cycloaddition of N-substituted saccharins 92 to nitrile imines 93 using *p*-HAP300 as a catalyst under solvent-free MW conditions to generate pyrazoline-containing N-substituted saccharins 94 (eqn (26)).^79^26
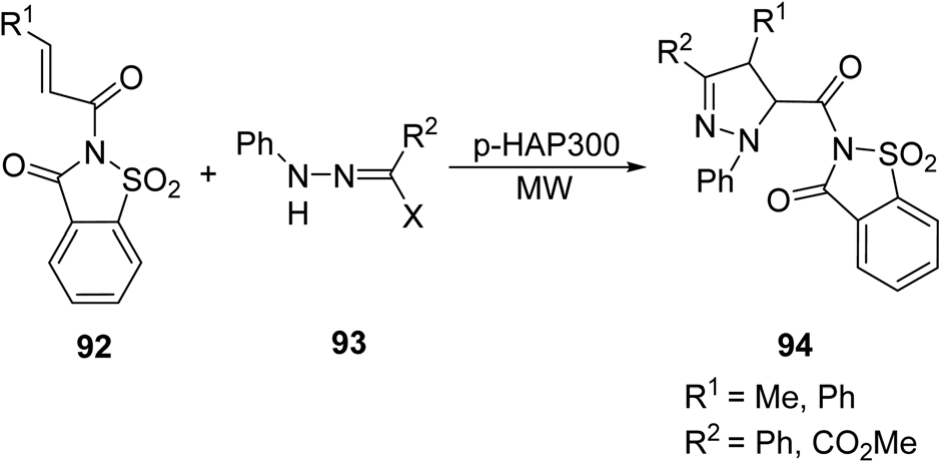


Four varieties of compound 94 were synthesized by conventional and microwave methods, and comparisons are shown in Table 8. Four variations of compound 94 were chosen as representative examples. The benefits to microwave synthesis in this case are colossal, with up to a 144-fold decrease in reaction time and an 80% increase in yield.

**Table 8:** Comparison of conventional and microwave methods in the synthesis of pyrazoline-containing N-substituted saccharins. Microwaves were ran at 115–142 °C^79^

Compound	R_1_	R_2_	Reaction time (min)		Yield (%)	
			Conventional	Microwave	Conventional	Microwave
94a	Me	Ph	660	6	10	90
94b	Me	CO_2_Me	480	6	10	89
94c	Ph	Ph	1440	10	8	87
94d	Ph	CO_2_Me	1440	10	5	80

Alam *et. al.*^80^ studied the solid state, MW-assisted synthesis of steroidal pyrazolines 96 through a one-pot reaction of α,β-unsaturated steroidal ketone 95 with thiosemicarbazide (eqn (27)). Heterocyclic embedded steroids offer medical chemists hybrid targets in the modular development of new and effective therapeutics; specifically, the pyrazoline unit can be a moderate nucleophilic target in the development of potentially bioactive steroids. MW protocols reduced the reaction times by a large factor (5 h to 3 min), with comparable or better yields.27
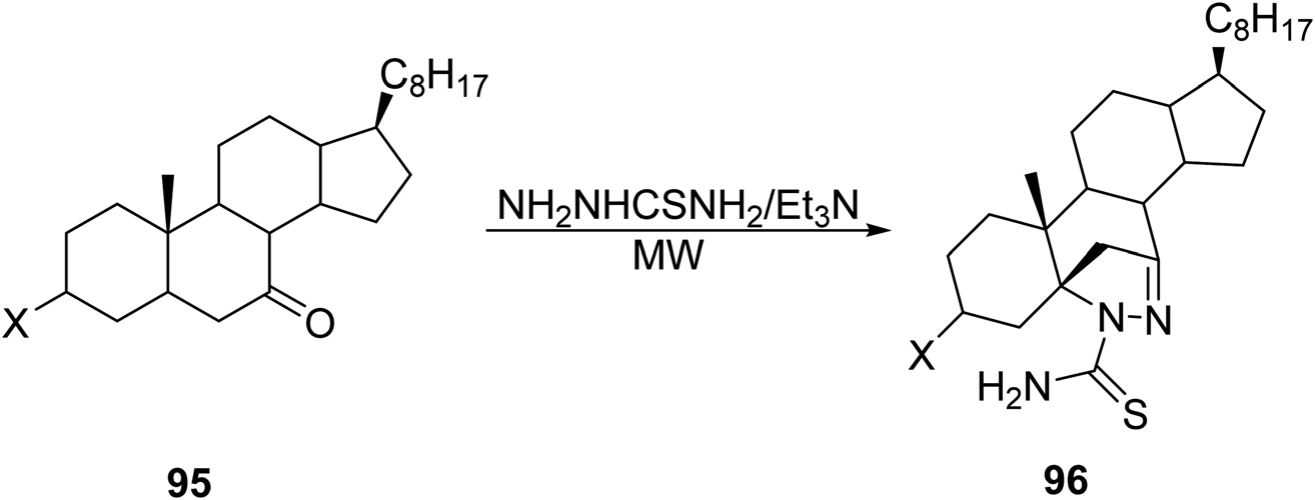


### Medicinal applications

Pyrazolines are gaining popularity in the pharmaceutical chemistry for their high versatility in various biological applications. They are widely incorporated into the structures of several compounds like antibacterial, antifungal, antidiabetic, antioxidant, antitubercular, anti-inflammatory, antitumor, anesthetic, analgesic and insecticidal agents. They also find use in the treatment of Parkinson's and Alzheimer's.^81^ They have shown activity against Gram positive (*S. aureus*, *M. luteus*) and Gram negative (*E. coli*, *S. typhi*) bacteria,^82^ but a lot of further compounds have been synthesized in order to avoid the common drug resistance.^83^ Substituted pyrazolines appended with different substituents were also tested against a yeast strain (*C. albicans*) showing good antifungal activity. Recently, thymol-based pyrazoline derivatives were synthesized by Raghuvanshi *et. al.* and tested for their antimalarial activity. A number of analogues were found to possess high antimalarial activity and show promise for future studies and agents.^84^ Furthermore, this unit is a valid pharmacophore, used as a lead compound for the synthesis of selective monoamine oxidase (MAO) inhibitors.^85^ 1, 3 and 5 are commonly substituted positions of the ring for the introduction of various aromatic and hetero-aromatic groups toward the development of new drugs for MAO-inhibition. A related consequence to this activity is its use as a scaffold with preeminent antidepressant and anticonvulsant properties in animal models.^86^

## 9. Lactam

Lactams are widely used in a number of areas ranging from drug discovery to the polymer industry.^87^ Preparation of lactams has long been an important topic in organic chemistry and continues to be actively pursued. Industrial synthesis of most lactams involves stepwise reactions using aggressive chemicals and high temperatures.^88^ However, MW protocols might be better alternatives that can be performed under milder reaction conditions and result in higher yields.

The chemistry that occurs involves the nucleophilic attack of a species (serine reside hydroxyl group) on the ring-strained lactam ring. The bicyclic β-lactam ring in amoxicillin is especially prone to hydrolytic cleavage of the amide to the secondary amine and ester, thus disrupting the normal cellular processes and allowing the cell to die.

**Scheme 6:**
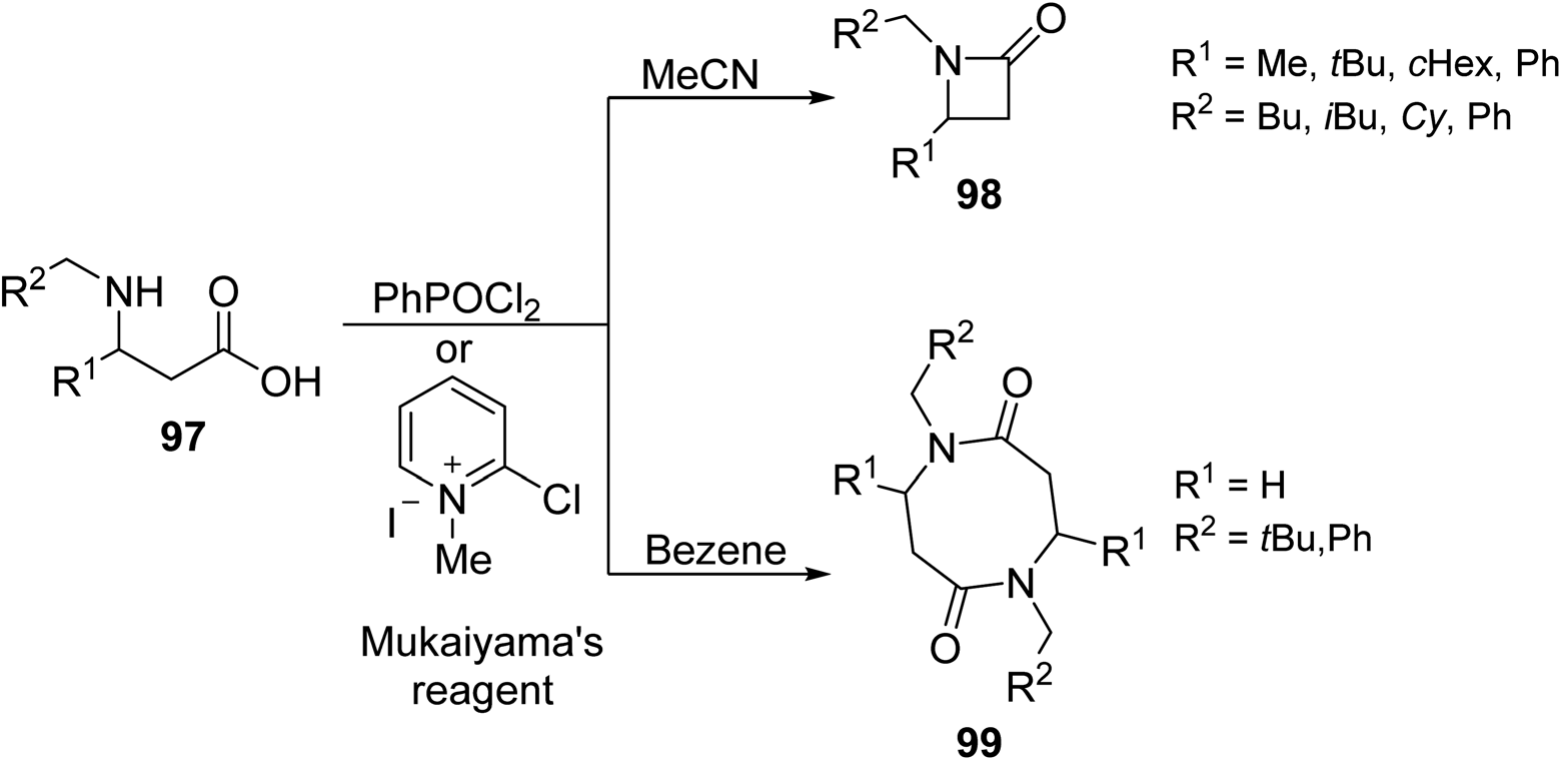
Synthesis of N-substituted-β-amino lactam derivatives.^89^

Hernandez-Vàzquez *et. al.*^89^ reacted several β-amino acids 97 through the activation of the C=O group with the organophosphorus reagent phenyl-phosphonic dichloride (PhPOCl_2_) or Mukaiyama's reagent under MW heating to prepare β-lactams 98 or cyclo-β-dipeptides 99 (Scheme 6). With PhPOCl_2_ (in benzene), the reaction followed two possible pathways depending on the β-substituent; when Mukaiyama's reagent is used, N-substituted-β-amino acids form either β-lactams or cyclo-β-dipeptides, or a mixture of both, just by changing the solvent used. In benzene, cyclo-β-dipeptide formation is favored, whereas acetonitrile affords β-lactam. The selectivity of the following reaction as a function of the solvent when Mukaiyama's reagent is used under MW irradiation may be a thermal effect. Considering that benzene and acetonitrile are both nonpolar solvents that do not readily absorb MW irradiation, the heating of the reaction under MW is directly determined by the polarity of the involved reagent. Mukaiyama's reagent, a pyridinium salt, can interact with MWs through ionic conduction, selectively heating the reaction; a sort of “molecular radiator” effect, where microscopic hot spots are created in the reaction vessel which can improve the selectivity of the chemical reaction.

**Scheme 7:**
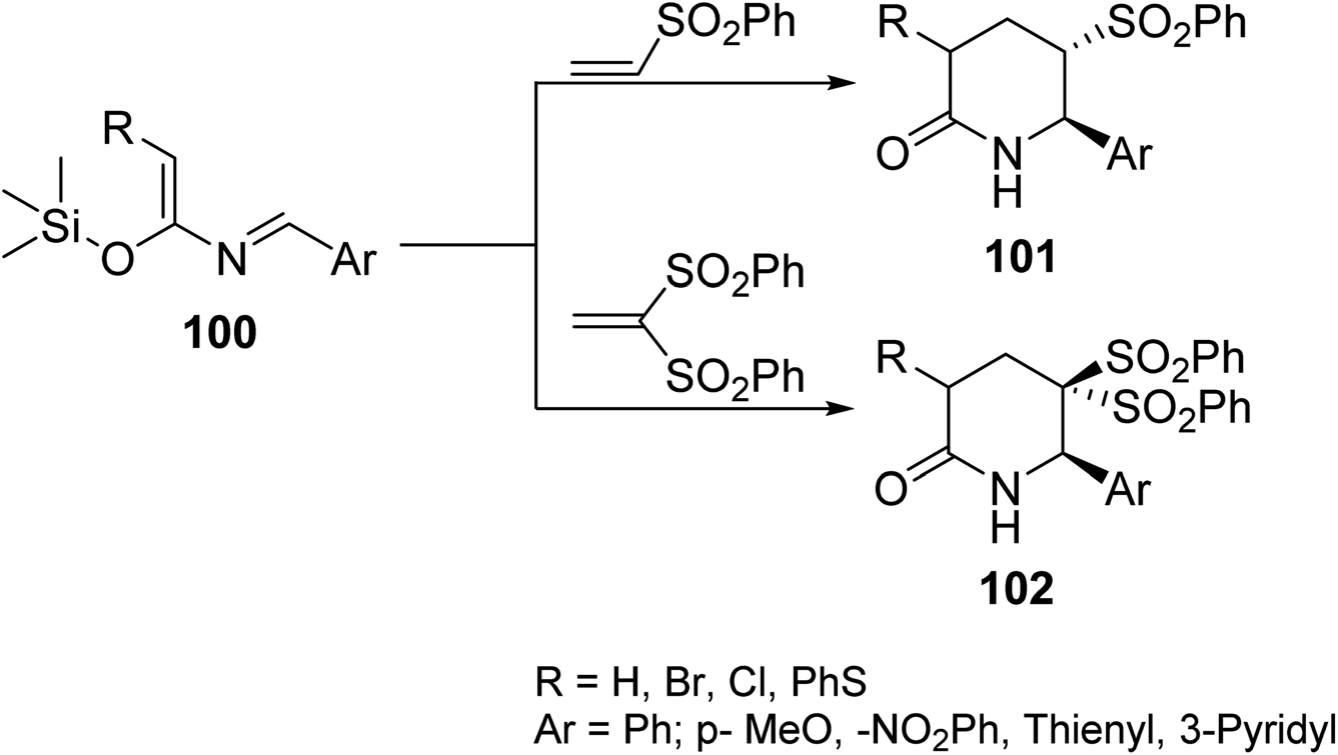
Synthesis of six-membered sulfonated lactam derivatives *via* azadiene.^90^

Long *et. al.*^90^ reported a hetero-Diels–Alder reaction of azadiene 100 with vinylsulfone dienophile in anhydrous toluene at 110 °C under MW irradiation to generate six-membered sulfonated lactams 101 and 102 (Scheme 7). Sulfonated functionality is an attractive feature on the lactam unit. It can be readily removed or elaborated to more complex decorations in the design of biologically important analogs.

δ-Glyconolactams 104 were prepared in good yields by Chen *et. al.*^91^ through a MW-assisted intramolecular Schmidt–Boyer reaction of δ-azido sugars 103 in trifluoroacetic acid, TFA (eqn (28)). δ-Glyconolactams are important class of compounds that not only have shown promise as glycosidase inhibitors, but also as potential precursors to many antibiotics.28
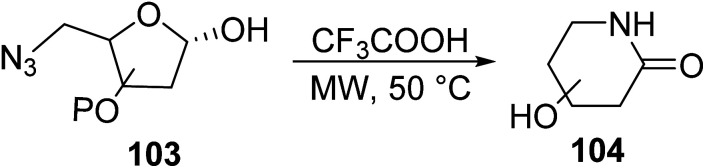


**Scheme 8:**
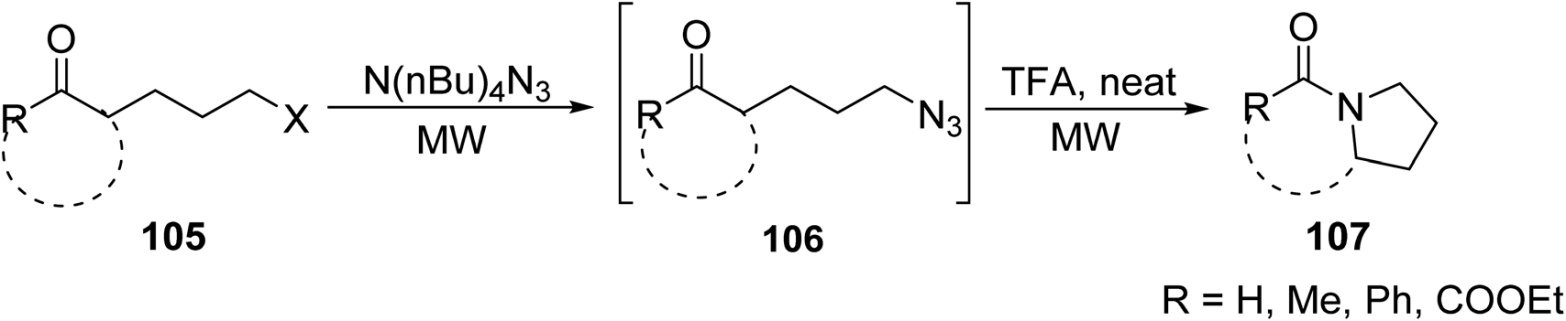
Synthesis of fused cyclic lactam derivatives *via* keto chloride.^92^

Painter *et. al.*^92^ reported the synthesis of a novel class of lactam analogs featuring fused cyclic/aryl moieties through a two-step MW heated flow reaction of keto chloride 105 with *N*,*N*,*N*,*N*-tetrabutylammonium azide (TBAA), to form intermediate 106, which subsequently cyclized in the presence of TFA to yield lactams 107 in good yield (Scheme 8). The combination of MW and flow chemistry allowed for the translation of the reaction into slightly bigger scale than the previous examples (0.05 g to 5 g).

Guérin *et. al.*^93^ described the homogeneous ring-closing metathesis reaction (RCM) of various fluoroalkene substrates 108 in the presence of a ruthenium complex under MW irradiation to give six- and five-membered fluorinated lactam rings 109 (eqn (29)). MW irradiation promoted higher yield in comparison to the conventional method. Yields were relatively lower for the more strained five-membered rings compare to their six-membered counterparts. Fluorinated lactams represent attractive targets in organofluorine chemistry that can be further converted into bioactive heterocycles.29
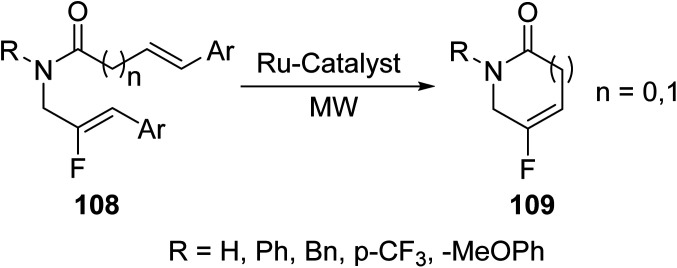


Two variations of compound 109 were used as representative examples. Although the benefits to using a microwave are not as high in lactam synthesis as they are in the synthesis of other heterocycles, the synthesis of compound 109 is made much more environmentally friendly by microwave synthesis (Table 9).^93^

**Table 9:** Comparison of conventional *versus* microwave techniques for the synthesis of compound 109. The microwave was ran at 120 °C^93^

Compound	R	Reaction time (min)		Yield (%)	
		Conventional	Microwave	Conventional	Microwave
109a	Bn	30	15	98	88
109b	PMB	30	15	100	100

### Medicinal applications

Well known is the antibiotic effect of β-lactams. They function by effectively preventing d-alanyl-d-alanine carboxypeptidases from catalyzing the biosynthesis of a peptidoglycan layer within the bacterial cell wall, resulting in arrested bacterial division. Due to the structural similarity of d-alanyl-d-alanine terminus, β-lactam antibiotics can irreversibly acrylate this transpeptidase, resulting in the interruption of biosynthesis and acceleration of bacterial cell wall hydrolysis. Despite the large number and efficacy of β-lactam compounds on the market, the increasing drug resistance among pathogenic bacteria claims the need for new antibiotic compounds. Besides their most notable use as antibiotics, substitutions on the pharmacophore groups make them versatile molecules with a broad range of medicinal uses,^94^ such as thrombin inhibitors, anti-hyperglycemic agents, anti-cancer agents, anti-proliferative agents, anti-HIV agents, fluorescent probes,^95^ antimalarial agents,^96^ anti-fungal agents, anti-tubercular agents, antioxidants, antimalarial agents and anti-fungal agents. A further development of β-lactams is their conversion to spirocyclic nuclei that show antidiabetic, anti-inflammatory, analgesic, anticancer and peptidomimetic properties. They can also inhibit acetyl-CoA cholesterol acyl transferase and picornaviruses.^97^ Recently, a combination of d-serine with β-lactam antibiotics was tested for activity against *Staphylococcus aureus* and showed promising results.^98^

## 10. 1,2,3-Triazole

In recent decades, the structure–activity relationship of triazoles has been studied and while variations can occur on the first, third, and fifth positions of the triazole moiety, the largest changes in activity occur when substitutions are made on the first position. This being said, the triazole moiety can hold a large number of substituents, making it an extremely useful group for pharmaceutical drugs.^99^

1,2,3-Triazoles are among the most widely used molecular moiety for drug synthesis and can be combined with a number of other functional groups, including those discussed above, to develop compounds that are useful for various medical functions. Thus, in the search for novel medicinal compounds, microwave synthesis is highly effective in creating new 1,2,3-triazole-containing compounds with high yields, low reaction times, and environmentally friendly methods.^100^

**Scheme 9:**
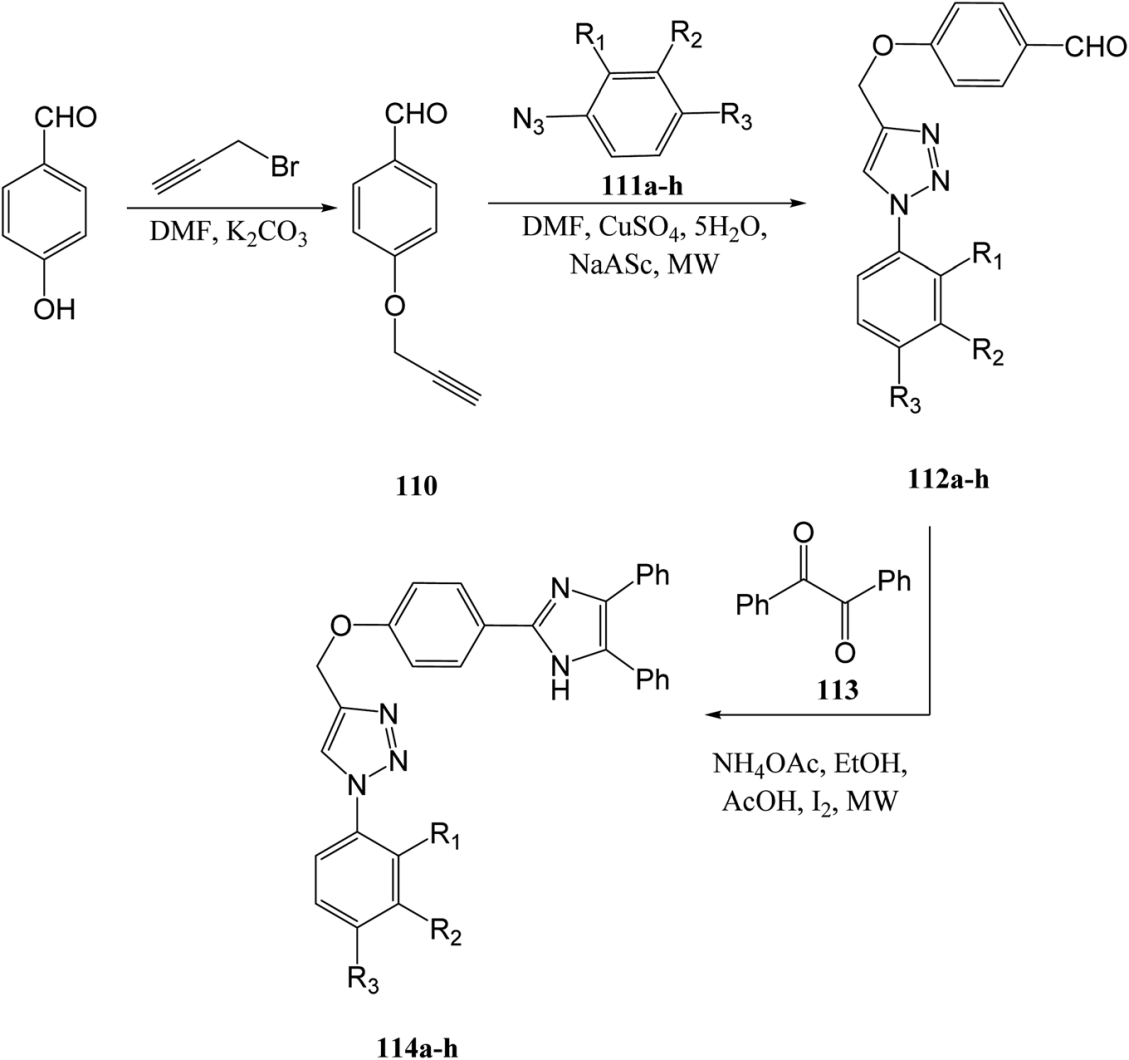
Synthesis of imidazole containing 1,2,3-triazole derivatives *via* 4-hydroxy benzaldehyde.^100^

N. J. P. *et. al.*^100^ created imidazole linked 1,2,3-triazole compounds in an effort to produce novel antimicrobial and antioxidant agents. 4-Hydroxy benzaldehyde was proporgylated to form compound 110 and then underwent click chemistry with various aryl azides 111a–h under microwave irradiation to induce triazole ring formation. Compounds 112a–h were then reacted with benzil 113 and ammonium acetate under microwave conditions to yield the target compounds 114a–h (Scheme 9).^100^

Amine *et. al.*^101^ synthesized compounds containing acridone and 1,2,3-triazole groups by copper(i)-catalyzed azide–alkyne cycloaddition under conventional and microwave conditions. This was achieved by reacting 10-(prop-2-yn-1-yl)acridone 115 and 2-azido-*N*-phenylacetamide 116 with copper sulfate and sodium ascorbate under various solvent and heating conditions to form the target compound 117 (eqn (30)).^101^30
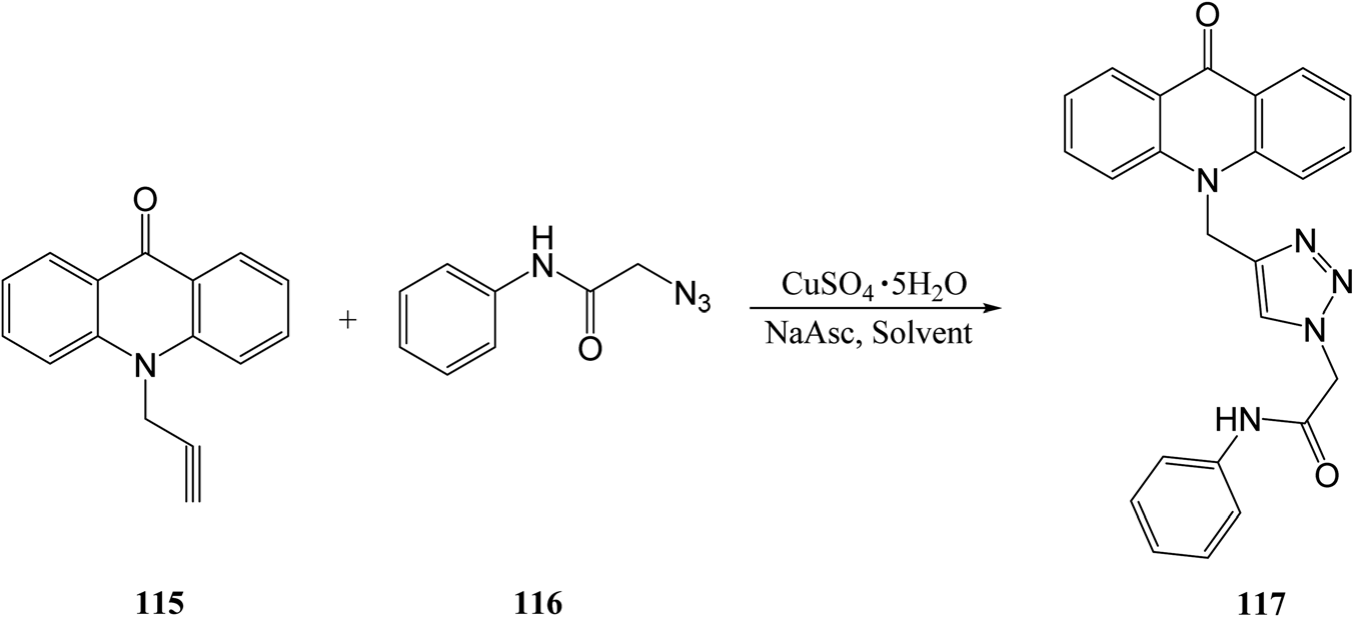


Compound 117 is used as another representative example to compare conventional *versus* microwave methods for the synthesis of 1,2,3-triazoles derivatives. Conventional synthesis consisted of stirring the reaction mixture at room temperature, while microwave-assisted synthesis consisted of reacting the mixture under microwave conditions at 200 W and 40–100 °C. As can be seen, microwave-assisted synthesis results in reaction times as low as 1.04% that of conventional methods. Additionally, microwave methods result in yields as much as 11% higher than those of conventional methods, as shown in Table 10.^101^

**Table 10:** Conventional *versus* microwave methods for the synthesis of target compound 117^101^

Solvent	Reaction time (min)		Yield (%)	
	Conventional	Microwave	Conventional	Microwave
*t*-BuOH/H_2_O	1440	15	55	60
*t*-BuOH	1440	15	55	60
DMF	600	10	73	81
DMF/H_2_O	1440	15	61	72
CH_2_Cl_2_	1440	15	68	71

As shown in eqn (31), Jayaram *et. al.*^102^ synthesized a disubstituted 1,2,3-triazole by reacting phenyl acetylene, sodium azide, and benzyl bromide with copper apatite and water. Conventional methods were conducted at 100 °C for 1.5–6 h, while microwave methods were conducted at 80 °C and 120 W for 5–20 min.^102^31
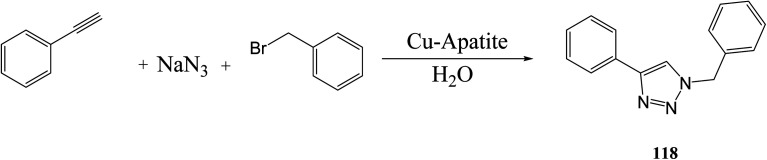


**Scheme 10:**
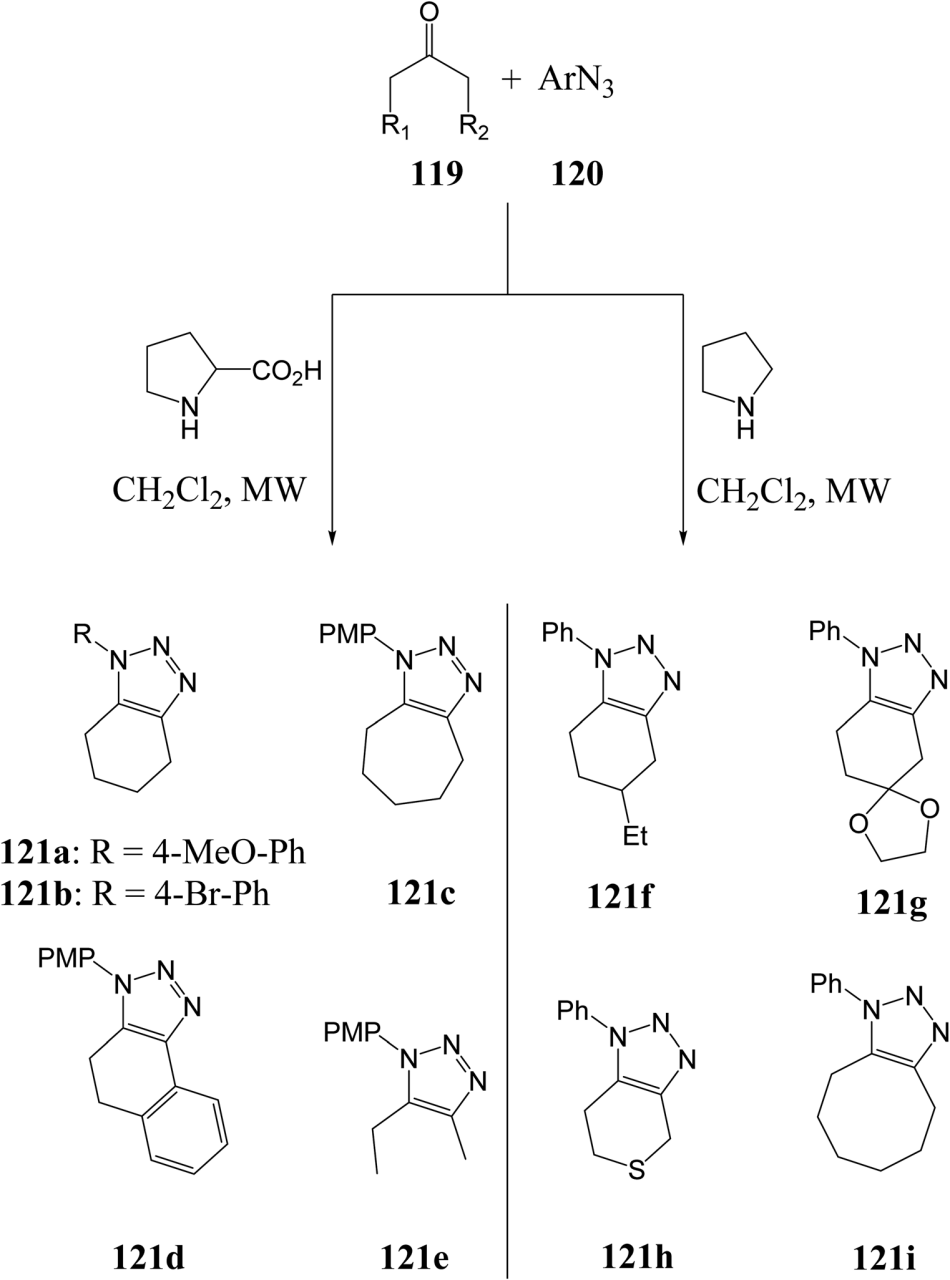
Synthesis of 1,2,3-triazole derivatives *via* unactivated ketones.^103^

Pons–Bressy *et. al.*^103^ used a proline catalyst to develop substituted 1,2,3-triazoles from unactivated ketones, while Wang *et. al.*^104^ used a pyrrolidine catalyst to synthesize 1,2,3-triazoles from unactivated ketones.^105^ The reactions were conducted under microwave irradiation at 80 °C in a dichloromethane solvent (Scheme 10).^105^

As depicted in eqn (32), Steenackers *et. al.*^106^ synthesized a library of 36 2-amino-1*H*-imidazole/triazole conjugates that can be used for anti-biofilm activity. The 2-hydroxy-2,3-dihydro-1*H*-imidazo[1,2-*a*]pyrimidin-4-ium salt 122 was reacted with phenylacetylene with a copper source and hydrazine hydrate in an ethanol and water solvent. Microwave-assisted synthesis was used, with a 35 W power and 90–100 °C temperature.^106^ The synthesized products showed moderate to high inhibitory activity against biofilms of *S. Typhimurium*, *P. aeruginosa*, *E. coli* and *S. aureus*.^106^32
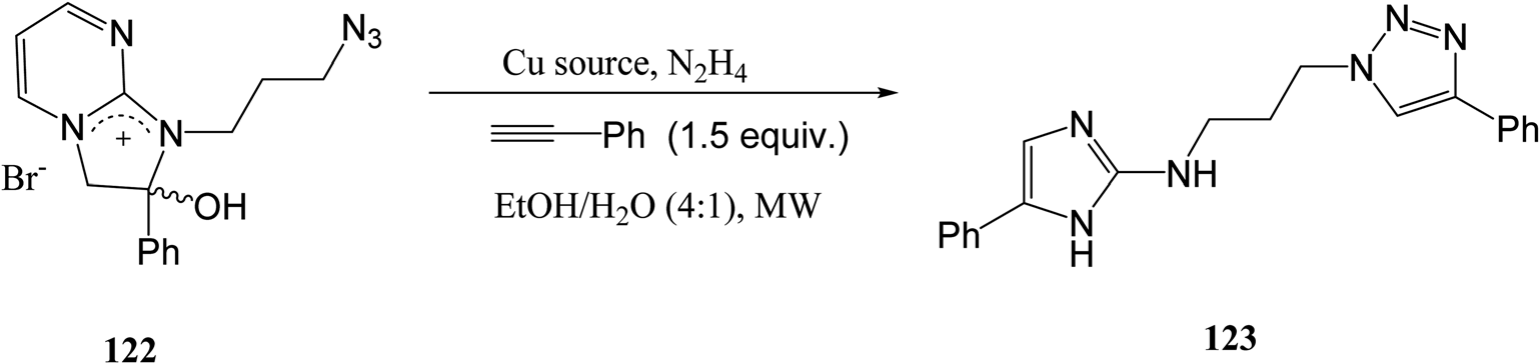


### Medicinal applications

**Fig. 9:**
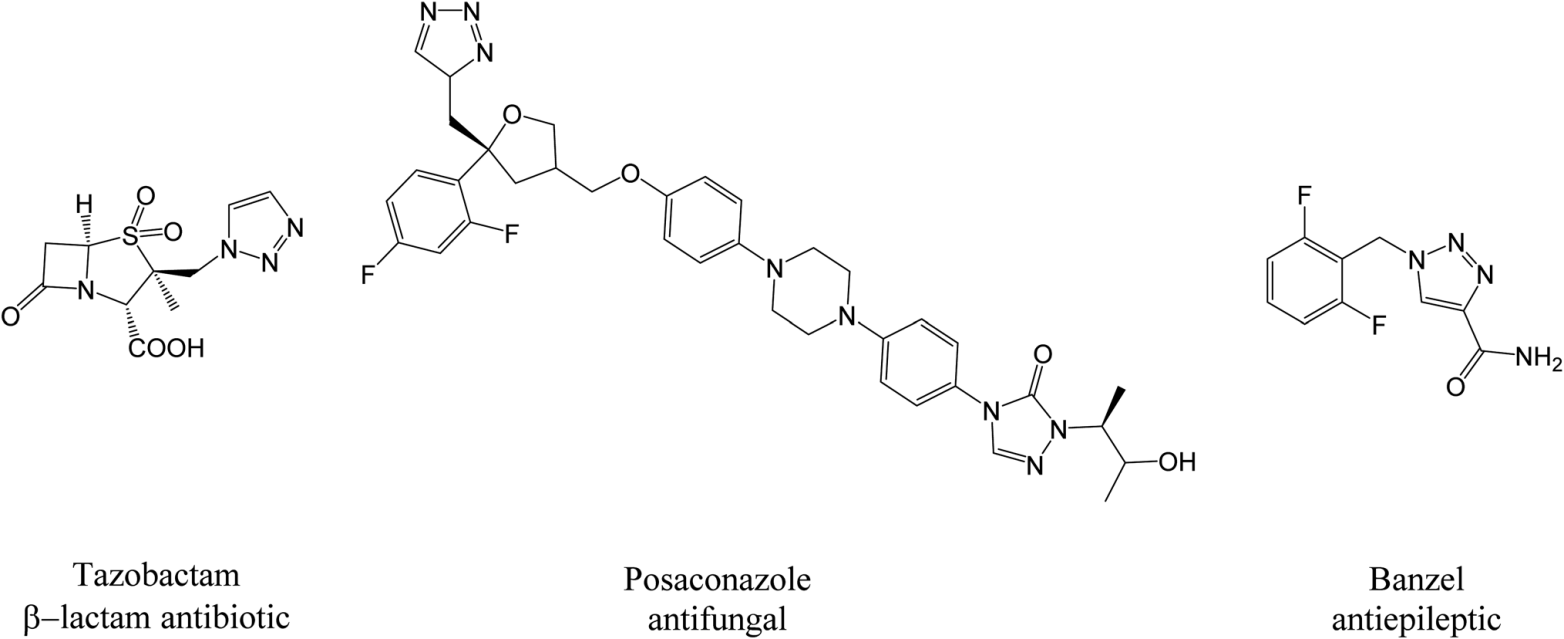
1,2,3-triazole containing pharmaceutical agents.

Substituted triazoles are, in many cases, incorporated into molecules that exhibit medicinal assets, which include antiproliferative, anticonvulsant, antimicrobial, antineoplastic, antiviral, analgesic, anti-inflammatory, anticancer, and antimalarial properties.^107^ In particular, a melampomagnolide B-triazole was found to be effective towards leukemia, melanoma, ovarian, and breast cancer cell lines.^108^ It is further known that 1,2,3-triazole moieties have distinct abilities against wild bacterial photogenes, and 1,2,3-triazoles with pyrimidine-chloroquinolines have exhibited high antiplasmodial activity against *P. falciparum*.^108^ Poulson *et. al.*^109^ has synthesized 4-[4-(4-methylphenyl)-1*H*-1,2,3-triazol-1-yl]benzenesulfonamide, which has shown promise as an obesity agent by decreasing lipogenesis, which it does by inhibiting human mitochondrial carbonic anhydrase isoenzymes.^99^ A number of 1,2,3-triazole containing drugs (Fig. 9) are already available for clinical use and have shown success in a number of areas, such as β-lactam antibiotics (Tazobactam),^110^ antifungal activity (Posaconazole),^99^ and antiepileptic activity (Banzel).^99^

## 11. Conclusion

The need to improve reaction conditions in the synthesis of organic heterocyclic compounds is an important, current topic that has promoted the quest for methodologies that will alleviate the many hurdles encountered. N-heterocycles are often the main structures of pharmaceutical compounds such as antibacterial, antifungal, anticancer and anti-depressive agents, but their synthesis can be complicated, resulting in several byproducts. MAOS clears some obstacles by enabling the selective manipulation of reaction parameters for the formation of many compounds in shorter reaction times with higher yields and purity that either do not occur under conventional heating or occur at much higher temperatures. This article highlights some key applications of MW-assisted synthesis in the ring formation of small and medium N-heterocycles and their medicinal uses. The many benefits of MW-assisted protocols showed to be the technique of choice in heterocycle chemistry. The examples cited in this review are impressive and provide a valuable insight to the application of MAOS as it pertains to diversifying some important nitrogen containing scaffolds.

## Conflicts of interest

There are no conflicts to declare.

## Supplementary Material
